# Effects of a Mindfulness Meditation App on Subjective Well-Being: Active Randomized Controlled Trial and Experience Sampling Study

**DOI:** 10.2196/10844

**Published:** 2019-01-08

**Authors:** Kathleen Marie Walsh, Bechara J Saab, Norman AS Farb

**Affiliations:** 1 Department of Psychological Clinical Science University of Toronto Scarborough Toronto, ON Canada; 2 Mobio Interactive Inc Biomedical Zone St. Michael's Hospital Toronto, ON Canada; 3 Preclinical Laboratory for Translational Research into Affective Disorders Psychiatric Hospital University of Zurich Zurich Switzerland; 4 Neuroscience Center Zurich University of Zurich and ETH Zurich Zurich Switzerland; 5 Department of Psychiatry, Psychotherapy and Psychosomatics Psychiatric Hospital University of Zurich Zurich Switzerland; 6 Department of Psychology University of Toronto Mississauga Mississauga, ON Canada

**Keywords:** mindfulness, attention, mobile health, interoception, mood, stress, psychological

## Abstract

**Background:**

Mindfulness training (MT) includes a variety of contemplative practices aimed at promoting intentional awareness of experience, coupled with attitudes of nonjudgment and curiosity. Following the success of 8-week, manualized group interventions, MT has been implemented in a variety of modalities, including smartphone apps that seek to replicate the success of group interventions. However, although smartphone apps are scalable and accessible to a wider swath of population, their benefits remain largely untested.

**Objective:**

This study aimed to investigate a newly developed MT app called Wildflowers, which was codeveloped with the laboratory for use in mindfulness research. It was hypothesized that 3 weeks of MT through this app would improve subjective well-being, attentional control, and interoceptive integration, albeit with weaker effects than those published in the 8 week, manualized group intervention literature.

**Methods:**

Undergraduate students completed 3 weeks of MT with Wildflowers (n=45) or 3 weeks of cognitive training with a game called 2048 (n=41). State training effects were assessed through pre- and postsession ratings of current mood, stress level, and heart rate. Trait training effects were assessed through pre- and postintervention questionnaires canvassing subjective well-being and behavioral task measures of attentional control and interoceptive integration. State and trait training data were analyzed in a multilevel model using emergent latent factors (acceptance, awareness, and openness) to summarize the trait questionnaire battery.

**Results:**

Analyses revealed both state and trait effects specific to MT; participants engaging in MT demonstrated improved mood (*r*=.14) and a reduction of stress (*r*=−.13) immediately after each training session compared with before the training session and decreased postsession stress over 3 weeks (*r*=−.08). In addition, MT relative to cognitive training resulted in greater improvements in attentional control (*r*=−.24). Interestingly, both groups demonstrated increased subjective ratings of awareness (*r*=.28) and acceptance (*r*=.23) from pre- to postintervention, with greater changes in acceptance for the MT group trending (*r*=.21).

**Conclusions:**

MT, using a smartphone app, may provide immediate effects on mood and stress while also providing long-term benefits for attentional control. Although further investigation is warranted, there is evidence that with continued usage, MT via a smartphone app may provide long-term benefits in changing how one relates to their inner and outer experiences.

**Trial Registration:**

ClinicalTrials.gov NCT03783793; https://clinicaltrials.gov/ct2/show/NCT03783793 (Archived by WebCite at http://www.webcitation.org/75EF2ehst)

## Introduction

### Background

Mindfulness training (MT) is a collection of meditation, introspection, and yoga practices aimed at the cultivation of psychological resilience and the alleviation of mental health symptoms [1]. In its modern secular form, MT was originally developed as an instructor-facilitated clinical group intervention for chronic pain and mood disorders [2,3], and much of its scientific efficacy stems from the study of these clinical interventions [4]. However, MT has recently been offered through a growing variety of novel and largely unvalidated delivery vehicles, including a growing number of smartphone apps. To date, there are no actively controlled experience sampling studies investigating whether such apps can replicate the therapeutic efficacy associated with validated group interventions.

Mindfulness has been defined as “the awareness that emerges through paying attention on purpose, in the present moment, and nonjudgmentally to the unfolding of experience moment by moment” [5]. Accordingly, MT aims to cultivate this adaptive form of awareness, primarily through guided meditation practices, suggesting that mindful awareness is a regulatory skill that can be developed over time [6]. To promote mindful regulation, mindfulness meditation has been integrated into a variety of MT interventions such as mindfulness-based cognitive therapy (MBCT) and mindfulness-based stress reduction (MBSR) [4]. Meta-analyses focusing on clinical populations have found moderate effects of mindfulness-based interventions on reducing symptom burden in chronic pain, anxiety, and depression [4,5,7]. In nonclinical populations, mindfulness-based interventions have been found to have strong effects on psychological well-being, including the reduction of stress, negative emotions, and anxiety [8]. Moreover, in both clinical and nonclinical populations, mindfulness-based interventions have been found to increase self-reported mindfulness [9,10]. Mindfulness meditation, both guided and self-guided, without the broader context of an MT intervention, has also been associated with improvements in well-being, including increases in self-reported mindfulness, improvements in attention, decreases in anxiety, decreases in stress, and reductions in negative personality traits [8,11].

Some of the proposed mechanisms for the effectiveness of MT include increases in metacognitive awareness, acceptance, and attentional control [12,13]. Metacognitive awareness involves being able to step back from one’s internal experiences and observe them from a third person perspective [14]. Acceptance involves a willingness to allow difficult internal experiences to happen while taking a nonjudgmental stance toward them; it has been suggested that greater acceptance reflects decreased experiential avoidance, which is attempting to change or control difficult internal experiences [15-17]. Attentional control may involve different subcomponents of attention, including the ability to direct attention toward stimuli (orienting), the ability to remain receptive to stimuli (alerting), and the ability to prioritize attention (conflict monitoring) [13]. These proposed mechanisms reflect key components of mindfulness, as defined by Bishop and colleagues, which includes self-regulation of attention and adopting an open and accepting attitude toward internal experiences [6].

Despite well-established benefits of mindfulness-based interventions, and some understanding of the mechanisms involved, MT dissemination can be difficult. For example, MBCT and MBSR require a commitment of weekly meetings and at-home practice of learned mindfulness skills for 8 weeks [3,18,19]. Moreover, these interventions are costly and not easily accessible because of the requirement of therapists to implement these interventions [20,21]. These limitations have prompted research on the minimum dose required for efficacious MT, and there is now some evidence that brief MT as short as 3 days to 4 weeks may have positive effects on anxiety, negative mood, mindfulness, perceived stress, and attention [22-24]. Moreover, a systematic review found no relationship between hours spent in MT sessions and changes in psychological distress [25], suggesting that formal meditation time is not the most important factor in efficacious MT. Indeed, a recent dismantling study of internet-based MT found no effect of formal meditation practice, although both formal and nonformal practice arms of the study outperformed a no-intervention control group [26].

Growing awareness of MT-related benefits, coupled with uncertainty around the necessary components leading to these benefits, has allowed for a rapid expansion of MT delivery modalities, including implementation through technological platforms. Technology-delivered mindfulness-based interventions have proven to be successful in improving well-being [27-29], including reductions in anxiety, depression, and stress [20,30-36]. Moreover, a variety of mindfulness-based smartphone apps have been developed that seek to replicate the success of group interventions [37]. However, although smartphone apps are scalable and accessible to a wider swath of population, their benefits remain largely untested [38].

Perhaps the fastest growing market for MT lies in smartphone apps for MT; the most popular current MT app, Headspace, boasted over 6 million users in 2016 [28]. However, despite a booming user base, only 4 randomized controlled trials have investigated the efficacy of smartphone apps for MT, and only half of these trials used an active control group. Van Emmerik and colleagues investigated the beneficial effects of a mindfulness app called VGZ Mindfulness Coach. After 8 weeks of using this app, participants demonstrated increases in mindfulness, improvements in psychiatric symptoms, and improvements in quality of life, relative to a waitlist control condition [21]. Similar findings were observed with the Headspace meditation app with regard to psychiatric symptoms; after using the Headspace app for 10 days, participants demonstrated reduced depressive symptoms and increases in positive affect, relative to an active control condition (participants had to make a list of what they did on that day the previous week). However, there were no changes in satisfaction with life or in negative affect. The authors reasoned that these findings may be a result of the short period that this app was used and that the changes in positive affect may have eventually led to changes in these other domains [39]. Two more recent randomized controlled trials have also investigated Headspace; the first trial found that after 10 sessions with Headspace, participants in the MT group demonstrated reductions in irritability and improvements in affective balance, relative to a psychoeducation control condition [40]. The second recent trial found that compared with a waitlist control, participants who completed 8 weeks of MT with Headspace demonstrated improvements in well-being and reductions in workplace stress [41].

Although these studies found some benefits from using these MT apps, they relied solely on subjective self-reports, which may be confounded with participant expectancy. For example, participants may believe that MT improves attention regulation [13], but such regulation can and should be assessed through behavioral performance rather than self-report alone. Moreover, these studies investigated the effects of MT while only comparing longitudinal *trait* outcomes, without evaluating the local or *state* effects of meditation sessions. Exploring state effects may be useful in demonstrating the immediate benefits of MT by limiting retrospective bias [42].

### Goal and Hypotheses

With few investigations of the effectiveness of MT apps on well-being, further research is warranted. The goal of this study was to better evaluate the local and longitudinal effects of app-delivered MT, relative to a randomized active-control group. For this purpose, we employed a newly developed MT app that was designed to collect user’s ratings of current mood and stress level as well as heart rate before and after each guided meditation session. In the active control condition, a popular cognitive game was adapted to allow for the same collection of mood, stress, and heart rate data. To investigate subtle changes across domains related to optimal psychological experience and functioning, a broad definition of well-being was measured, including both hedonic (ie, pleasure vs pain) and eudemonic aspects (ie, realizing one’s true nature) [43], and a data-driven approach was used to efficiently report on these domains.

As outcome variables, we attempted to provide several longitudinal and local MT targets. For longitudinal targets, we modeled 3 commonly cited MT benefits: improved subjective well-being, attentional control [8-11,13], and interoceptive integration [44-47]. For local targets, we tested for improvements in mood, physiological arousal [24,48,49], and stress [11,22,26,50].

It was hypothesized that MT via a smartphone app would improve trait subjective well-being, attentional control, and interoceptive integration, albeit with weaker effects for a brief 3 weeks of MT with the app than those published in the 8-week manualized group intervention MT literature. In addition, it was expected that beneficial state MT effects would be observed in mood, heart rate, and perceived stress, suggesting the immediate benefits of brief mindfulness meditation.

## Methods

### Recruitment and Design

Undergraduate students were recruited from the University of Toronto Mississauga and randomly assigned to train with 1 of 2 smartphone apps: Wildflowers, an MT app or 2048, a cognitive training app, which was used as an active control condition to control for expectancy and daily engagement. Both apps were described to participants as a cognitive training app that might promote well-being. This description was given to foster positive expectancy in the active control condition, without introducing any real stressor or emotion regulation training.

To be eligible to participate in this study, participants were expected to (1) have normal or corrected-to-normal vision and hearing, (2) be 18 years or older, (3) be fluent in English, and (4) own an iPhone, iPad, or iPod with access to the internet.

Upon recruitment, each participant was asked to come in to the laboratory to complete self-report questionnaires of well-being through a Web-based survey platform called Qualtrics and complete behavioral measures of attentional control and interoceptive integration on a computer in the laboratory. After completing the questionnaires and tasks, participants downloaded their assigned app and made sure it was working on their phone and they knew how to use it. Participants did not know their condition assignment until after completing the pretraining measures. Ratings of current mood, stress level, and heart rate were recorded within each app before and after each training session. Heart rate was sampled with the camera on the participants’ smartphone using a well-established algorithm. This technique included an internal reliability check where if reliability was low, heart rate data were not provided to the user or researchers [51-53]. After 3 weeks of training, using their assigned app for at least 10 min per day, each participant returned to the laboratory to retake the self-report questionnaires and behavioral measures of attentional control and interoceptive integration.

Before participating in the study, undergraduate students gave written informed consent. Participants were aware that their usage data (date and usage time, mood, stress, and heart rate) from each of the apps was sent anonymously via email to the researchers. Students recruited through the university’s undergraduate recruitment site received course credit for their participation. Students recruited via flyers posted throughout the university received Can $10 for every hour spent in the laboratory and for using their assigned app, to a maximum of Can $90 in compensation for their participation. The research protocol was approved by the University of Toronto Social Sciences, Humanities, and Education Research Ethics Board (REB). This study was retrospectively registered on ClinicalTrials.gov; ID: NCT03783793.

### Training Conditions

#### Mindfulness Training App

Mindfulness training in the study was performed using a new app called Wildflowers (Mobio Interactive Inc, Toronto), which was developed in collaboration with our laboratory. This smartphone app incorporates features that have been deemed to be important to include in smartphone MT, as suggested by Mani and colleagues [37]. For example, Wildflowers includes guided meditations such as breathing, body scans, and open monitoring practices and also provides didactic content in the form of lessons and information about the benefits of MT. In addition, the app was designed to collect user’s ratings of current mood and stress level as well as heart rate, before and after each guided meditation session. This feedback is aggregated and provided to the user and might be useful in providing the user with helpful insights into the physiological and psychological benefits of MT.

Using the Wildflowers app (Multimedia Appendix 1), participants were able to choose and complete a variety of guided meditations. Participants could decide on a certain mindfulness meditation through different avenues. First, they could complete a lesson on a certain type of meditation (eg, mindfulness of breath or mindfulness of body). Each lesson included (1) a fact about the particular meditation; (2) teaching the user about *snapshots* to record current mood, stress level, and heart rate; (3) a minute of flow where the participant was asked to connect with the present moment; (4) the meditation; (5) a fact on how to increase resilience such as practicing being nonjudgmental; and (6) ending with another snapshot. Instead of a lesson, participants could also choose from a library of guided meditations that are each unlocked after completing a certain number of meditations. Finally, participants could also have a guided meditation suggested to them based on their current mood and stress level.

The Wildflowers MT app is freely available in the Apple App Store and on Google Play, with additional content and features available to subscribing customers. The training experience described in this study is available through the free features on the app.

#### Cognitive Training With 2048

The training app for the control condition was based on an open source code for a popular cognitive training app called 2048, which is marketed by Ketchapp in the Apple app store as a “fun and relaxing puzzle game” (Multimedia Appendix 2). Within 2048, participants slide numbered tiles around a grid, matching tiles of the same value. Instead of tiles disappearing, as in *Candy Crush* or other similar grid-sliding games, matching 2 numbered tiles in 2048 combines them into 1 new tile displaying the sum of the previous 2 numbers. For example, two 2-tiles linked side-by-side become a 4-tile, whereas 2 matched 4-tiles become an 8-tile, and so on. The goal is to match tiles until the sum of 2048 is reached on a single tile. There is no time limit. Importantly, the identical in-app psychobiometric features for ratings of mood, stress, and heart rate before and after each training session were built into the control condition app to provide parity in measurement of state effects between the 2 training conditions.

### Measures of Subjective Well-Being

#### Perceived Stress Scale

The Perceived Stress Scale (PSS) [54] is a 10-item scale that measures the global perception of stress. However, because of a question that was inadvertently missing when the 10-item PSS questionnaire was loaded onto the survey platform, Qualtrics, participants from both groups did not see or respond to this missing question during data collection. Therefore, results from the short 4-item version of the PSS were alternatively used in subsequent analyses. The 4-item PSS has demonstrated satisfactory evidence of internal consistency and convergent validity [55].

#### Big Five Inventory

The Big Five Inventory (BFI) [56,57] is a 44-item scale that measures the 5 dimensions of personality: extraversion, agreeableness, conscientiousness, neuroticism, and openness. Extraversion includes sociability, assertiveness, and positive emotionality. The BFI has demonstrated excellent evidence of internal consistency, test-retest reliability, and convergent validity [57,58].

#### Psychological Well-Being Scale

The Psychological Well-Being Scale (PWBS) [59] is an 84-item questionnaire that measures psychological well-being. This measure includes 6 subscales measuring autonomy, self- acceptance, positive relations with others, environmental mastery, purpose in life, and personal growth. The PWBS has demonstrated satisfactory evidence of internal consistency [59] and convergent validity and excellent evidence of test-retest reliability [60].

#### Acceptance and Action Questionnaire-II

The Acceptance and Action Questionnaire-II (AAQ-II) [17] is a 7-item scale that measures psychological inflexibility and experiential avoidance. The AAQ-II has demonstrated satisfactory evidence of internal consistency and excellent evidence of test-retest reliability and convergent validity [17].

#### Philadelphia Mindfulness Scale

The Philadelphia Mindfulness Scale (PHLMS) [61] is a 20-item scale that measures 2 components of mindfulness: awareness and acceptance. The PHLMS has demonstrated satisfactory evidence of internal consistency and convergent validity [61]. However, test-retest reliability has not been reported [62].

#### Multidimensional Assessment of Interoceptive Awareness

The Multidimensional Assessment of Interoceptive Awareness (MAIA) [63] is a 32-item scale that measures the multidimensional construct of interoceptive body awareness. This scale is made up of 8 subscales: noticing, not distracting, not worrying, attention regulation, emotional awareness, self-regulation, body listening, and trusting. The MAIA has demonstrated satisfactory evidence of convergent validity, internal consistency [63], and test-retest reliability [44].

#### Spiritual Experience Index-Revised

The Spiritual Experience Index-Revised (SEI-R) [64] is a 23-item scale that measures a person’s faith and spiritual journey. This scale consists of 2 subscales: the spiritual support subscale and the spiritual openness subscale. The SEI-R has demonstrated satisfactory evidence of convergent validity and excellent evidence of internal consistency [64]. However, test-retest reliability has not been reported.

#### Meaning in Life Questionnaire

The Meaning in Life Questionnaire (MLQ) [65] is a 10-item scale that measures 2 dimensions of the meaning in life and as such includes 2 subscales: presence of meaning and search for meaning. The MLQ has demonstrated satisfactory evidence of internal consistency, convergent validity, and test-retest reliability [65].

#### Mood Board Circumplex

The mood board is a visual representation of negative and positive emotions on a spectrum, ranging from intense emotions to mild emotions. This mood board provides a maximum of 32 emotions that a participant can select and yields 4 scores: degree of intense negative emotions, degree of intense positive emotions, degree of mild negative emotions, and degree of mild positive emotions. This questionnaire is currently under validation; however, the words chosen for the mood board are commonly used in other measures of mood [66,67]. In addition, previous research has demonstrated the efficacy in taking these emotion-specific measures of mood and converting them to a visual analog scale with 4 dimensions [68].

For additional details and psychometric properties for each of the questionnaires used in this study, please see Multimedia Appendix 3.

### Measure of Attentional Control

#### Centre for Research on Safe Driving-Attention Network Test

The Centre for Research on Safe Driving-Attention Network Test (CRSD-ANT) is a 10-min version of the Attention Network Test (ANT) that measures 3 different functions of attention: alerting, orienting, and conflict monitoring [69]. Alerting involves achieving and maintaining attention to incoming stimuli, orienting involves directing attention to sensory input, and conflict monitoring involves resolving conflict among responses [70]. This behavioral task requires participants to determine whether a directional object (car) is pointing left or right, and the network scores (alerting effect, orienting effect, and conflict effect) are calculated as the difference between median response times [69,70].

### Measure of Interoceptive Integration

#### Respiration Integration Task

The Respiration Integration Task (RIT) is a newly developed behavioral task created in our laboratory to assess interoceptive attention (see Multimedia Appendix 4 for rationale and validity evidence). In the RIT, participants view a circle on a computer screen that expands and contracts rhythmically. In each trial, participants will view 2 cycles of expansion and contraction, the reference and the target. The reference circle always expands and contracts at a fixed rate, whereas the target varies in its frequency. Participants are to report on whether the target is faster or slower than the reference. The change in the frequency of cycling begins with a large change (about 2000 ms) and employs a psychophysics staircase to determine the *just noticeable difference* of change detection. The staircase uses a *3 up/1 down* algorithm in which 3 consecutive correct responses reduce the frequency change in the subsequent trial, making it more difficult, whereas 1 incorrect response increases the frequency difference, making it easier.

The RIT has 3 phases, a vision only baseline, a respiration entraining practice period, and the respiration integration period. During the baseline, participants use vision alone to detect changes in circle frequency. Once this threshold is established, participants spend 60 seconds entraining their breath, that is, practicing matching respiration to the movement of the circle as it pulses at the reference frequency. Afterwards, in the integration period, participants repeat the task while matching their breathing to the expansion and contraction of the sphere. The visual and breath scores are calculated by taking the mean frequency across the final 6 trials from each of these conditions.

### Statistical Analysis

#### Power

An *a priori* power analysis for the group-specific training effects was modeled as the interaction of the within-subjects factor of time (pre vs post) and the between-subjects factor of group (MT vs control). The power analysis was conducted using the G*Power software app to determine how much power would be needed to find weak-to-moderate interaction effects in this study. A moderate effect, eta-squared of 0.06 or Cohen *F* of 0.25, was assumed. It was also assumed that repeated measures scores had a moderate-to-strong correlation of .5. The analysis suggested a total N=34 for 80% power. A weaker effect of Cohen *F*=0.15 would require 90 participants, and so the study was powered conservatively for this effect, that is, we attempted to recruit approximately 45 participants in each group.

Following data analysis, a post hoc power analysis simulation, with 10,000 simulations, was conducted using the statistical platform R 3.4.3 [71] to more accurately simulate the post hoc power of the study. Scores were assumed to start at 0 and have an SD of 1 to detect a 0.5 (half deviation) change in the MT group and no true change in the control group, with an effect size *d*=0.5, which is considered moderate according to Cohen [72]. The simulation revealed this study (n=45 per group) had 65% power to detect the desired interaction effect. Using the simulation approach, the study would have needed a sample size of n=90 per group to achieve 80% power. The discrepancy between the G*Power and simulation approaches suggests a need for further research on power calculation methodology.

#### Data Exclusion

Participants were excluded from analysis if they did not adhere to the study protocol. Minimal adherence was defined as 10 min of practice per day, missing no more than 4 of the 21 days, and completing both the pre- and posttraining assessment measures.

#### Data Reduction

An exploratory factor analysis (EFA) was conducted on the scale measures listed above in the R statistical computing environment [71]. The number of factors required was first estimated using the paran library for performing Horn’s parallel analysis of principal components or factors [73].

#### Group Comparisons

All statistical analyses were conducted using the statistical platform R 3.4.3 [71], with an alpha level of .05 for all tests. Demographics between groups were compared using a *t* test and a chi-square test. Before group comparisons, the questionnaire data were reduced using EFA to increase ease of interpretability and minimize type I error. Multilevel models were used to compare both state and trait measures of well-being between groups over time. Finally, the relationship between the state and trait measures of well-being were investigated through correlations.

## Results

### Participants

**Figure 1:**
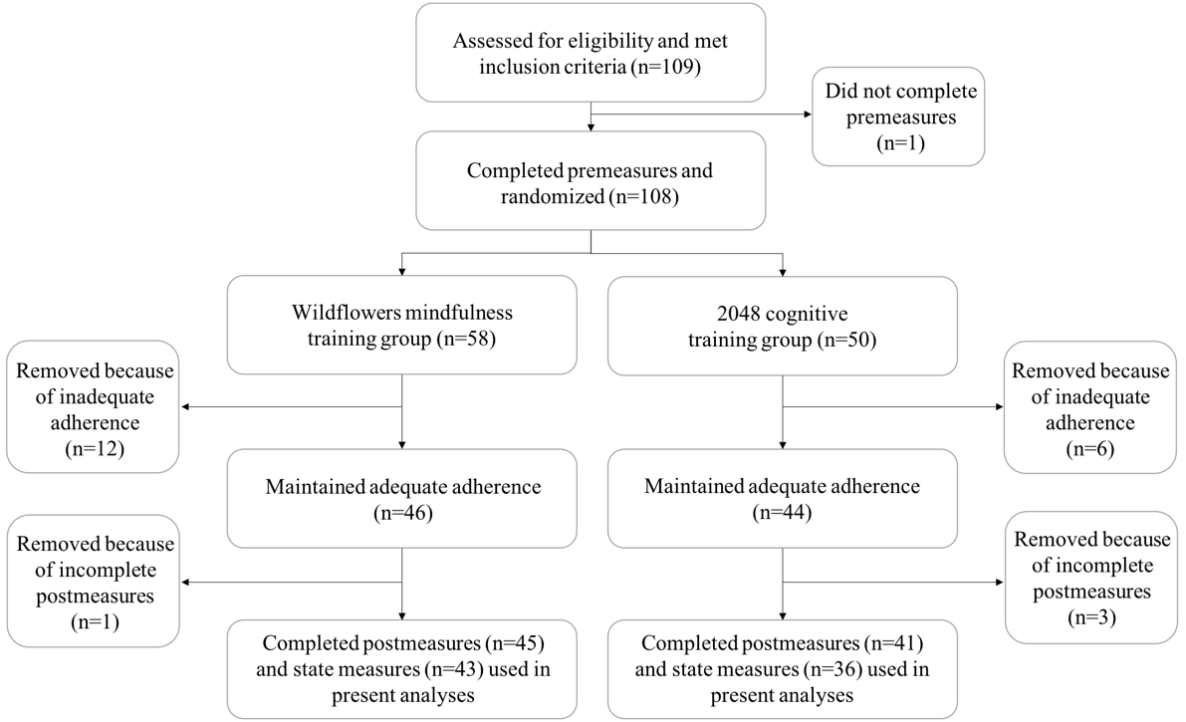
Participant flow diagram.

As shown in the participant flow diagram for the study (Figure 1), the final sample included 41 participants in the cognitive training group (mean age 19.78 [SD 2.43], 88% female) and 45 participants in the MT group (mean age 20.24 [SD 2.63], 80% female). A *t* test revealed that the groups did not significantly differ in terms of age (*t*_82.86_=−0.85, 95% CI −1.56 to 0.62; *P*=.40), and a chi-square test revealed that the groups did not significantly differ in terms of gender (χ^2^_2_=5.5, *P*=.06). On average, participants practiced a total of 16.32 days (cognitive training=16 days and MT=16.59 days), 20.21 sessions (cognitive training=19.54 sessions and MT=20.74 sessions), and 5.05 hours (cognitive training=4.46 hours and MT=5.57 hours).

### Statistical Analysis Assumptions

The data were inspected to make sure that assumptions that could affect the interpretation of the results were satisfied. Inspection of the normality of residuals, influential cases, autocorrelation of residuals, and homogeneity of variances revealed no major violation of assumptions (see Multimedia Appendix 5).

### Data Reduction

Before conducting the EFA, the factorability of the 31 questionnaire subscales in this study was examined. It was determined that all of the subscales were suitable to include in the EFA (see Multimedia Appendix 5). Horn’s parallel analysis of principal components [73] suggested that 4 factors should be retained in the EFA (Multimedia Appendix 6); however, as the fourth factor was well below the random eigenvalues generated during the analysis test, a 3-factor solution was chosen to be more suitable. The EFA was conducted using ordinary least squares to find the minimum residual solution using the psych package [74] in R, and an oblique rotation method, promax, was used to allow for correlations between factors.

The 3-factor solution (Table 1) explained 42.5% of the shared variance. It was determined that factor 1 (eigenvalue=6.45) was best labeled as acceptance, as this factor included subscales measuring acceptance and not avoiding or worrying about psychological discomfort. Factor 2 (eigenvalue=4.34) was best labeled as awareness because of the inclusion of subscales measuring psychological and physical awareness and attention regulation.

**Table 1:** Factor loadings of well-being questionnaires entered into the exploratory factor analysis.

Scale/subscale	Acceptance factor loadings	Awareness factor loadings	Openness factor loadings
PSS^a^-short version	−0.67^b^	0.06	−0.13
BFI^c^/Extraversion	0.38^b^	0.20	0.06
BFI/Agreeableness	0.29	0.32^b^	0.01
BFI/Conscientiousness	0.41^b^	0.04	0.26
BFI/Neuroticism	−0.65^b^	0.08	−0.06
BFI/Openness	−0.05	0.33	0.42^b^
PWBS^d^/Autonomy	0.50^b^	−0.01	0.38
PWBS/Environmental Mastery	0.76^b^	0.03	0.21
PWBS/Personal Growth	0.21	0.20	0.46^b^
PWBS/Positive Relations with Others	0.47^b^	0.25	−0.02
PWBS/Purpose in Life	0.65^b^	0.15	0.15
PWBS/Self-Acceptance	0.84^b^	−0.04	0.17
AAQ-II^e^	0.87^b^	−0.10	−0.01
PHLMS^f^/Awareness Subscale	−0.15	0.66^b^	0.20
PHLMS/Acceptance Subscale	0.73^b^	−0.30	−0.06
MAIA^g^/Noticing	−0.15	0.82^b^	−0.04
MAIA/Not Distracting	0.48^b^	−0.07	−0.23
MAIA/Not Worrying	0.35^b^	−0.17	0.26
MAIA/Attention Regulation	0.10	0.66^b^	0.08
MAIA/Emotional Awareness	−0.18	0.90^b^	0.02
MAIA/Self-Regulation	0.01	0.68^b^	0.11
MAIA/Body Listening	−0.04	0.66^b^	0.00
MAIA/Trusting	0.39	0.52^b^	0.09
SEI-R^h^/Support	0.05	0.24^b^	−0.11
SEI-R/Openness	0.19	0.12	0.36^b^
MLQ^i^/Presence of Meaning	0.59^b^	0.17	−0.13
MLQ/Search for Meaning	−0.40^b^	0.40	0.04
Mood Board/Intense Negative Emotions	−0.45	− 0.13	0.54^b^
Mood Board/Mild Negative Emotions	−0.56^b^	−0.12	0.47
Mood Board/Intense Positive Emotions	0.08	0.00	0.66^b^
Mood Board/Mild Positive Emotions	0.00	0.00	0.61^b^

^a^PSS: Perceived Stress Scale.

^b^Represents the strongest loadings for each latent factor.

^c^BFI: Big Five Inventory.

^d^PWBS: Psychological Well-Being Scale.

^e^AAQ-II: Acceptance and Action Questionnaire-II.

^f^PHLMS: Philadelphia Mindfulness Scale.

^g^MAIA: Multidimensional Assessment of Interoceptive Awareness.

^h^SEI-R: Spiritual Experience Index-Revised.

^i^MLQ: Meaning in Life Questionnaire.

Finally, factor 3 (eigenvalue=2.38) was best labeled as openness and included subscales measuring openness, personal growth, and the reporting of both negative and positive emotions. For the reliability analysis, a subscale was considered to be a part of a factor if its loading was greatest for that factor, relative to the other factors (values that show strongest loadings for each latent factor are shown in Table 1). Each of the factors demonstrated good evidence of internal reliability; the acceptance factor had an internal reliability of alpha=.89, the awareness factor had an internal reliability of alpha=.86, and the openness factor had an internal reliability of alpha=.70. In addition, acceptance and awareness (*r*=.32), acceptance and openness (*r*=.21), and awareness and openness (*r*=.35), each demonstrated a positive relationship with each other.

### Longitudinal Training Effects

#### Subjective Well-Being

To test the hypothesis that trait well-being would improve over time as a result of MT, each of the 3 factors (acceptance, awareness, and openness) were analyzed in a multilevel model using the nlme package [75] in R.

Each of the 3 factors from the EFA were modeled as a function of time (pre- vs posttraining) and group (MT vs cognitive training). In addition, pairwise follow-up comparisons, Tukey Honest Significant Difference test corrected for multiple comparisons, using least-squares means were conducted using the lsmeans function from the lsmeans package [76] in R.

Analysis of subjective well-being data revealed a significant main effect of time for the acceptance factor (Table 2 and Multimedia Appendix 7) as well as a trend toward an interaction between time and group. Follow-up comparisons suggested that this marginal interaction was driven by a significant increase in acceptance (lsmean difference −0.42 [SE 0.08]; *t*_84_=−5.02; *P*<.001) from pre- to posttraining for participants in the MT condition. In addition, a trend was observed where participants at postcognitive training had lower levels of acceptance than the participants at post-MT (lsmean difference −0.52 [SE 0.20]; *t*_84_=−2.56; *P*=.06).

A significant main effect of time was observed for the awareness factor (Table 2 and Multimedia Appendix 7). Follow-up comparisons revealed that from pre- to post-MT, participants demonstrated increased levels of awareness (lsmean difference −0.43 [SE 0.11]; *t*_84_=−3.98; *P*<.001). In addition, from pre- to postcognitive training, participants demonstrated increased levels of awareness (lsmean difference −0.30 [SE=0.11]; *t*_84_=−2.65; *P*=.046).

There was no main effect of time or interaction between time and group observed for the openness factor (Table 2 and Multimedia Appendix 7). A main effect of group was observed, suggesting that randomization failed to equate openness. However, the effects for the acceptance and awareness factors were maintained after controlling for openness in the earlier analyses.

Uncorrected multilevel models were conducted for each of the individual questionnaire subscales (Multimedia Appendix 8). The results from these multilevel models mirror the results observed for the acceptance, awareness, and openness latent factors, suggesting that these 3 factors are an accurate summary of the well-being questionnaires.

##### Attentional Control

To test the hypothesis that attentional control would improve as a result of MT, each of the 3 network scores from the CRSD-ANT (orienting effect, alerting effect, and conflict effect) were analyzed in a multilevel model. Each of the network scores were modeled as a function of time (pre- vs posttraining) and group (MT vs cognitive training). In addition, pairwise follow-up comparisons were conducted.

Analysis of the CRSD-ANT revealed no main effects or interactions for the alerting effect (Table 2 and Multimedia Appendix 7) or for the orienting effect (Table 2 and Multimedia Appendix 7).

**Figure 2:**
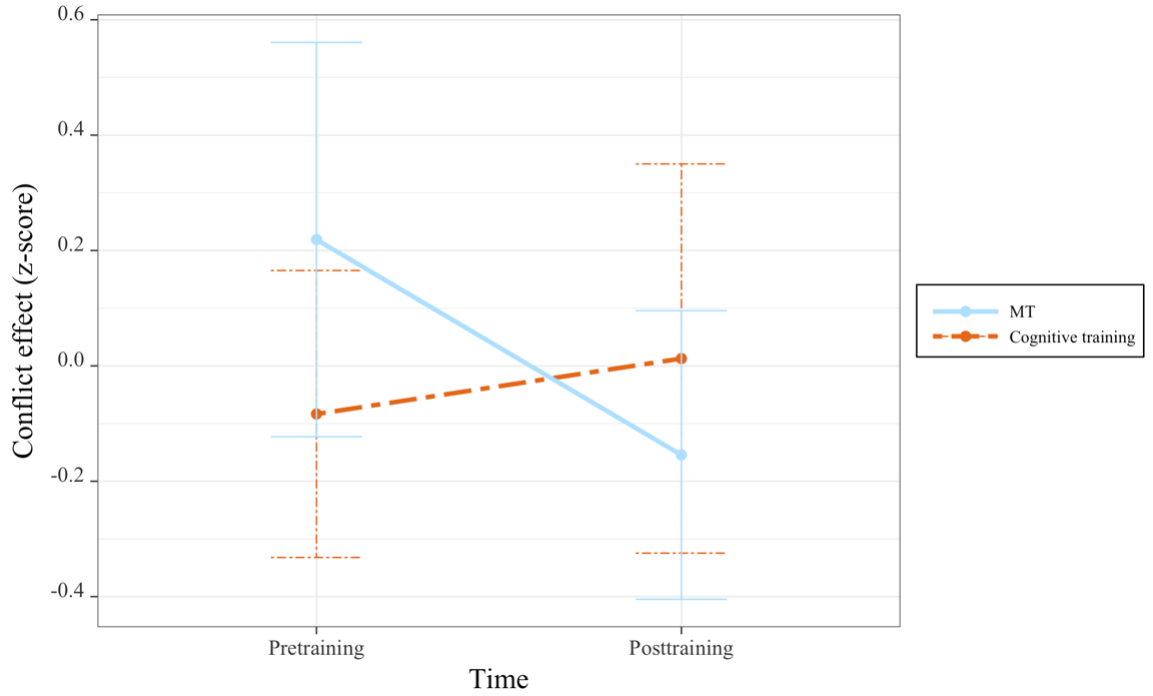
Changes in conflict effect before and after mindfulness training (MT) and cognitive training.

A significant interaction between time and group was observed for the conflict effect (Table 2 and Figure 2). Follow-up comparisons revealed that this interaction was driven by significant improvements in the conflict effect from pre- to posttraining for participants in the MT group (lsmean difference 0.37 [SE 0.14]; *t*_84_=2.63; *P*=.05), but there was no evidence of change in the active control group.

**Table 2:** Multilevel models of trait well-being measures.

Dependent and independent variable		Estimate (SE)	*t* value (*df*)	*P* value	Pearson *r* effect size
**Acceptance**					
	Time	0.19 (0.09)	2.12 (84)	.04^a^	0.23
	Group	0.28 (0.20)	1.40 (84)	.17	0.15
	Time×group	0.24 (0.12)	1.93 (84)	.06^b^	0.21
**Awareness**					
	Time	0.30 (0.11)	2.65 (84)	.01^a^	0.28
	Group	0.26 (0.20)	1.28 (84)	.20	0.14
	Time×group	0.13 (0.15)	0.83 (84)	.41	0.10
**Openness**					
	Time	0.04 (0.10)	0.43 (84)	.67	0.05
	Group	0.47 (0.19)	2.49 (84)	.01^a^	0.26
	Time×group	−0.07 (0.14)	−0.50 (84)	.62	−0.05
**Alerting effect**					
	Time	−0.03 (0.19)	−0.14 (84)	.89	−0.02
	Group	−0.09 (0.22)	−0.43 (84)	.67	−0.05
	Time×group	0.37 (0.26)	1.43 (84)	.16	0.15
**Orienting effect**					
	Time	−0.03 (0.18)	−0.18 (84)	.85	−0.02
	Group	−0.10 (0.22)	−0.45 (84)	.65	−0.05
	Time×group	0.36 (0.25)	1.43 (84)	.16	0.15
**Conflict monitoring**					
	Time	0.10 (0.15)	0.65 (84)	.52	0.07
	Group	0.30 (0.22)	1.40 (84)	.16	0.15
	Time×group	−0.47 (0.21)	−2.29 (84)	.02^a^	−0.24

^a^Represents significant findings.

^b^Represents marginal findings.

##### Interoceptive Integration

To test the hypothesis that behavioral interoceptive attention would improve as a result of MT, participants’ scores from the RIT were analyzed in a multilevel model, modeled as a function of group (MT vs cognitive training), time (pre- vs posttraining), and condition (visual baseline vs breath integration). In addition, pairwise follow-up comparisons were conducted.

**Figure 3:**
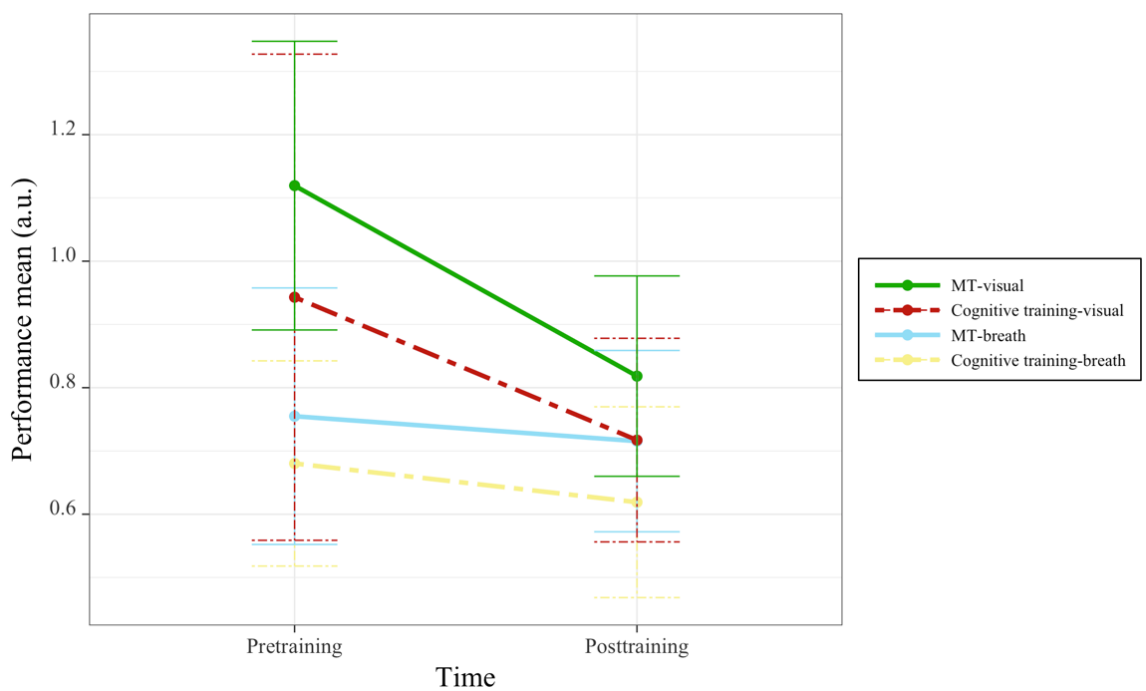
Changes in respiratory integration task performance by task condition, group, and time. MT: mindfulness training.

This analysis revealed a main effect of condition for the RIT, with the breath condition associated with better detection thresholds than the visual baseline condition (Table 3 and Figure 3). However, the results showed no indication of MT effects over time.

### State Training Effects

#### Subjective Well-Being

To test the hypothesis that participants in the MT group would demonstrate immediate effects on well-being, each of the in-app measures (mood, stress, and heart rate) were analyzed in a multilevel model. Each of these measures were modeled as a function of group (MT vs cognitive training), time (multiple training sessions per participant), and session (before vs after each training session), with subject, time, and session as random intercepts.

**Table 3:** Multilevel model of respiration integration task performance.

Independent variable	Estimate (SE)	*t* value (*df*)	*P* value	Pearson *r* effect size
Time	−0.06 (0.13)	−0.50 (213)	.62	−0.03
Group	0.09 (0.15)	0.57 (83)	.57	0.06
Condition	0.26 (0.12)	2.16 (213)	.03^a^	0.15
Time×group	0.02 (0.17)	0.12 (213)	.91	0.01
Time×condition	−0.16 (0.18)	−0.94 (213)	.35	−0.06
Group×condition	0.10 (0.17)	0.60 (213)	.55	0.04
Time×group×condition	−0.10 (0.24)	−0.41 (213)	.68	−0.03

^a^Represents significant findings.

**Figure 4:**
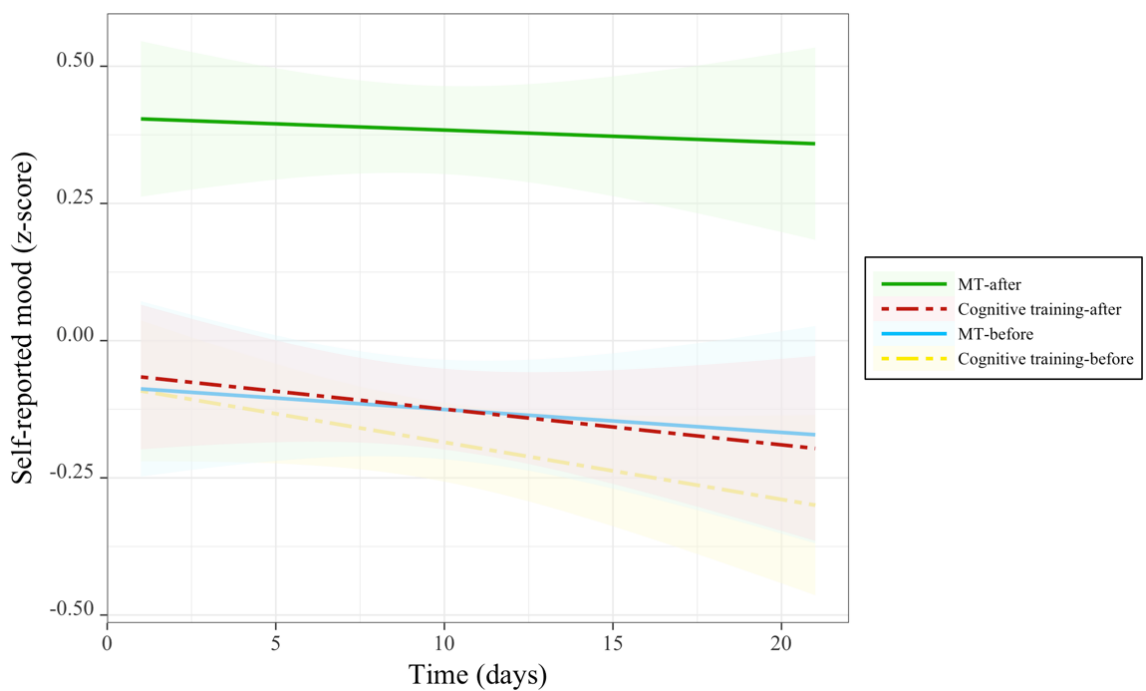
Changes in mood before and after each training session over the course of training. MT: mindfulness training.

Analysis revealed a significant interaction between group and session on mood (Table 4 and Figure 4). Follow-up comparisons revealed that participants in the MT group demonstrated a significant improvement in mood after each training session (lsmean difference −0.51 [SE 0.03]; *t*_1190_=−15.15; *P*<.001), whereas participants in the cognitive training group did not.

**Figure 5:**
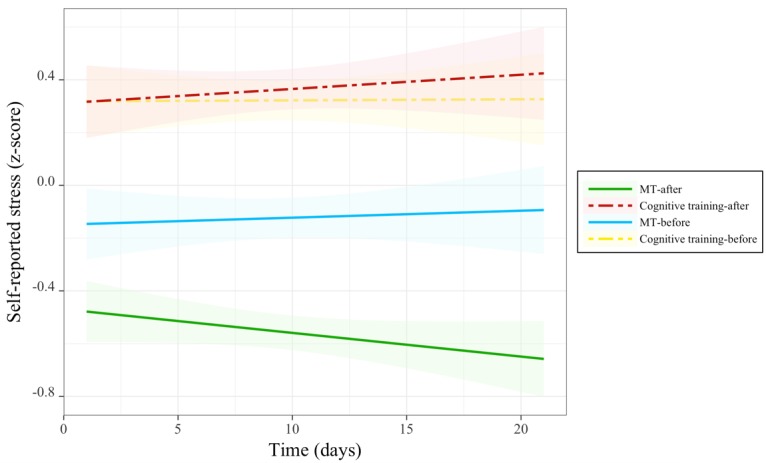
Changes in stress before and after each training session over the course of training. MT: mindfulness training.

A significant main effect of group; an interaction between group and session; and 3-way interaction between time, group, and session were demonstrated for ratings of stress level (Table 4 and Figure 5). Being a part of the MT group was generally associated with lower stress, even before practice sessions: follow-up comparisons revealed that participants in the MT group relative with the cognitive training group demonstrated significantly lower levels of subjective stress both in pretraining (lsmean difference 0.44 [SE 0.14]; *t*_76_=3.11; *P*<.01) and posttraining sessions (lsmean difference 0.91 [SE 0.14]; *t*_76_=6.42; *P*<.001). For the group by session interaction, follow-up comparisons revealed that participants in the MT group demonstrated a significant decrease in stress levels after each training session (lsmean difference 0.43 [SE 0.02]; *t*_1190_=17.96; *P*<.001), whereas participants in the cognitive training group did not. For the 3-way interaction, significant reductions of stress over time were uniquely observed for participants in the MT group posttraining session (beta=−0.01 [SE 0.004]; *t*_616_=−2.65; *P*<.01; *r*=−.11), but such time effects were neither observed pretraining in the MT group nor at pre- or posttraining for the cognitive training group. Together, these results indicate participants in the MT training group began daily training sessions with less overall stress, MT sessions uniquely produced a further reduction in stress, and the impact of training sessions in the MT group uniquely increased over the 3-week training period.

**Table 4:** Multilevel models of state measures of well-being.

Dependent variable and independent variable		Estimate (SE)	*t* value (*df*)	*P* value	Pearson *r* effect size
**Mood**					
	Time (days)	−0.01 (0.01)	−1.65 (1117)	.10	−0.05
	Group	−0.01 (0.15)	−0.07 (76)	.95	−0.01
	Session (pre vs post)	0.02 (0.07)	0.31 (1190)	.75	0.01
	Time×group	0.01 (0.01)	0.85 (1117)	.40	0.03
	Time×session	0.004 (0.01)	0.63 (1190)	.53	0.02
	Group×session	0.47 (0.09)	4.99 (1190)	<.001^a^	0.14
	Time×group×session	−0.002 (0.01)	−0.21 (1190)	.84	−0.01
**Stress**					
	Time (days)	−0.004 (0.01)	−0.75 (1117)	.45	−0.02
	Group	−0.55 (0.16)	−3.48 (76)	.001^a^	−0.37
	Session (pre vs post)	−0.01 (0.05)	−0.14 (1190)	.89	−0.004
	Time×group	0.01 (0.01)	1.43 (1117)	.15	0.04
	Time×session	0.01 (0.004)	1.13 (1190)	.26	0.03
	Group×session	−0.31 (0.07)	−4.66 (1190)	<.001^a^	−0.13
	Time×group×session	−0.02 (0.01)	−2.78 (1190)	.005^a^	−0.08
**Heart rate**					
	Time (days)	0.001 (0.01)	0.17 (1064)	.86	0.01
	Group	−0.08 (0.14)	−0.54 (75)	.59	−0.06
	Session (pre vs post)	−0.01 (0.09)	−0.10 (1067)	.92	−0.003
	Time×group	0.01 (0.01)	0.63 (1064)	.53	0.02
	Time×session	0.01 (0.01)	1.72 (1067)	.09	0.05
	Group×session	0.13 (0.13)	1.02 (1067)	.31	0.03
	Time×group×session	−0.03 (0.01)	−2.18 (1067)	.03^a^	−0.07

^a^Represents significant findings.

**Figure 6:**
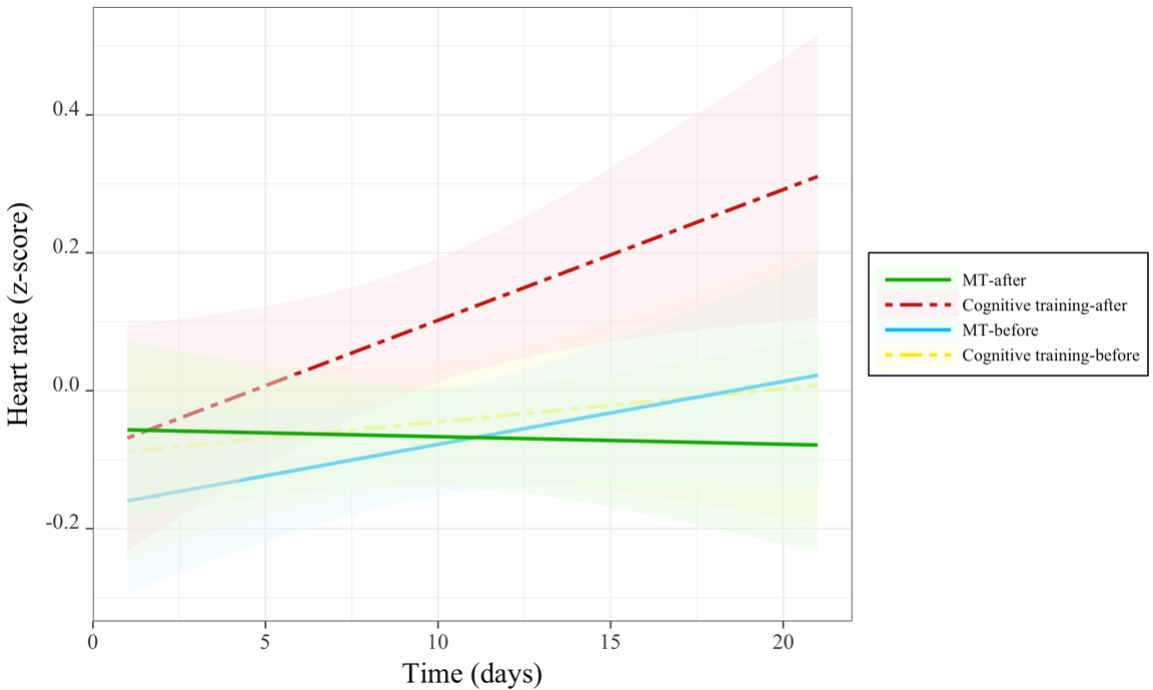
Changes in heart rate before and after each training session over the course of training. MT: mindfulness training.

A significant 3-way interaction between time, group, and session was observed for heart rate (Table 4 and Figure 6). Follow-up comparisons revealed that this interaction was driven by participants in the cognitive training group, for whom posttraining heart rate increased over the course of the training period (beta=−0.02 [SE 0.01]; *t*_1011_=−2.03; *P*<.04; *r*=−.06), whereas pretraining heart rate in the cognitive training group and both pre- and posttraining heart rate in the MT group did not change with time. These results suggest that the cognitive training became increasingly arousing in terms of heart rate over the study period, but no such effects were associated with MT.

### Association Between Trait and State Measures of Well-Being

An exploratory analysis of the associations between change scores for the trait measures (pre- and posttraining) and change scores for the state measures (pre- and postpractice session) were conducted via correlation analysis. Results (Table 5) revealed significant relationships between state and trait measures of well-being: changes in acceptance with changes in mood, changes in acceptance with changes in stress, and changes in orienting effect with changes in heart rate. In addition, there were significant relationships within trait measures such as changes in conflict effect with changes in acceptance and changes in orienting effect with changes in acceptance.

**Table 5:** Correlations between state (pre- and postsession) and trait (pre- and postintervention) measures of well-being change.

State and trait well-being	State well-being			Trait well-being					
	Heart Rate	Stress	Mood	Accept^a^	Aware^b^	Open^c^	Alerting	Orienting	Conflict
Stress	−.19								
Mood	−.17	−.34^d^							
Acceptance	−.18	−.34^e^	.42^d^						
Awareness	.01	−.17	.20	.25^e^					
Openness	.18	−.08	.19	.14	.40^f^				
Alerting	−.47^e^	.11	−.07	−.06	−.12	−.09			
Orienting	.06	−.22	.25	.22^e^	.02	−.01	.22^e^		
Conflict	.03	.11	−.10	.29^d^	−.04	−.01	.14	.13	
Group	−.33	−.61^f^	.57^f^	.21	.09	−.05	.15	.15	−.24^e^

^a^Accept: acceptance.

^b^Aware: awareness.

^c^Open: openness.

^d^*P* value<.01.

^e^*P* value<.05.

^f^*P* value<.001.

## Discussion

### Principal Findings

This was the first actively controlled study to investigate whether MT apps can promote the therapeutic effects associated with validated group MT interventions, namely, subjective well-being, attentional control, and interoceptive integration. A data-driven approach was used to allow for a broad canvassing of well-being, while also providing a parsimonious interpretation of observed changes in well-being. This approach yielded 3 latent factors: acceptance, awareness, and openness. The clear distinction between loadings onto an acceptance and awareness factor reflect the 2 subfactors of the PHLMS [61], suggesting that these latent variables provided an accurate summary of well-being domains associated with MT. In addition, the openness factor provided a new source of variability that is not commonly measured separately in a mindfulness study.

Subjective well-being was assessed both in terms of trait (pre- and posttraining) and state (pre- and postpractice session) self-reports. A trend toward MT-specific changes in acceptance from pre- to posttraining was observed, and closer inspection of the data suggested that the MT group might have driven a general increase in acceptance over time. This result was complemented by MT effects at the state level; relative to the cognitive training group, participants in the MT group demonstrated improved mood and reduced stress following each training session. Importantly, changes in acceptance across the intervention were correlated with session-specific changes in stress and mood. Although the overall effect of training on acceptance was weak, this is one of the first documented reports of state-effects of meditation contributing to interventional level effects on dispositional mindfulness.

These findings are consistent with a broader literature in which dispositional acceptance has been associated with reduced experiential avoidance [15-17], decreased negative affect, and reduced stress reactivity [77,78]. At the state level, brief mindfulness interventions have been linked to beneficial effects on stress and mood [24,48]. However, few studies have described how changes at the dispositional or trait level relate to individual training session effects. Here, we provide some of the first evidence that it is precisely these session-level effects on mood and stress appraisals that manifest as trait-like changes in distress tolerance. Specifically, it seems that app-guided MT may have immediate effects on mood and stress and that these effects help to explain broader changes in the self-appraised capacity to cope with negative experiences. Such a finding is in keeping with the principles of MT in which practitioners are taught to engage rather than avoid negative emotions and reduce their impact on more general mood and stress appraisals. Encouragingly, the beneficial impact of MT on subjective stress in the MT group increased over time. This effect is evidenced by a significant decline over the course of training in postsession stress levels for the MT group. Therefore, over a longer time course, accumulating state effects of MT practice may support greater changes in acceptance, especially with greater adherence to practice than what was observed in this study; however, further research is warranted to support this hypothesis.

Contrary to the study hypotheses, participants in the MT and cognitive training groups reported significant increases in both acceptance and awareness over the study period. One explanation for this finding may be the fact that participants in both groups recorded their mood and stress levels before and after each training session. Research has shown that recording mood and stress in and of itself may contribute to improvements in negative symptomatology by increasing emotional self-awareness [79] and could promote acceptance of negative emotion by exposing participants to the natural variation in daily affective experience. Both groups performed daily ratings on mood and stress before and after each training session, a reflective practice that could itself foster awareness and insight around emotional experience. Furthermore, the general increase in acceptance and awareness may help to explain why MT-specific increases in acceptance were so modest: change acceptance and awareness were moderately correlated and the active control group may have benefitted from the increased awareness inherent to a daily reflection study design. This result not only suggests a benefit to even minimal daily reflection on emotional experience but also supports the importance of including an active control group in contemplative research. Without such a control group, the increase in awareness in the MT group may have suggested that this change was related to the mindfulness component in the MT smartphone app. However, with the cognitive training group in the study, it was possible to further ascertain benefits unique to MT above and beyond general effects of the daily reflection paradigm.

There were no training effects for either group observed for the openness factor. This result is not entirely surprising in the context of research that has shown that those who choose to practice mindfulness demonstrate greater openness [80], and openness was not predicted a priori to emerge as a factor for analysis. In this study, participants in the MT group demonstrated overall greater openness than participants in the cognitive training group. However, openness did not appear to be impacted by training in either group, and controlling for individual differences in openness did not alter the other study findings. As participants were unaware of randomization condition at baseline assessment, it is unlikely that the group difference was caused by experimental condition and more likely reflects the difficulty in equating all study variables through random assignment.

Attentional control was assessed on a trait level using the CRSD-ANT, which yielded alerting, orienting, and conflict effect scores. Analyses revealed training effects specific to MT; relative to the cognitive training group, 3 weeks of MT led to greater improvements in conflict monitoring. However, training effects were not observed for alerting effect or orienting effect. These results are in line with Tang and colleagues [49] who measured attentional control using the 20-min version of the ANT and found that after 5 days of integrated body-mind training (IBMT), which included MT along with several other body-mind techniques, participants in the IBMT condition demonstrated improvements in executive functioning relative to the relaxation group. In addition, no differences in orienting effect or alerting effect were found. Similarly, Zeidan and colleagues [23] found improvements in executive functioning after 4 days of MT relative to an active control group, and Ainsworth and colleagues [81] found improvements in executive function after focused attention and open monitoring MT, relative to a control group. The present results are also reflected in studies comparing naïve meditators with experienced meditators, which have found that experienced meditators demonstrate greater cognitive flexibility [82-84]. Taken together, the results of this study suggest that using an MT app may provide similar benefits as other MT interventions for increasing attentional control and cognitive flexibility.

Conflict monitoring, also known as executive attention or switching [85], is a form of attention regulation that includes self-regulation (cognitive, emotion, and behavior) [85,86]. In this study, improvements in conflict monitoring observed in the MT group may reflect improved self-regulation skills, and indeed, changes in conflict monitoring scores were moderately correlated with changes in acceptance. Improved self-regulation skills have been associated with improvements in trait mindfulness [87], which in this study may be evidenced by the significant positive correlation observed between conflict monitoring and acceptance. Moreover, previous research has found that greater emotional acceptance may mediate the effects of MT on executive control [88]. Although here both the MT and cognitive training groups demonstrated an equivalent increase in acceptance, with a larger sample or dose of MT, it is possible that MT-specific enhancement in conflict monitoring may promote later MT-specific increases in acceptance.

Interoceptive attention was assessed with the respiration integration task. In terms of interoceptive attention, there were no training effects. However, participants in both groups demonstrated greater accuracy when using their breath to judge the circle rather than just using their visual abilities. These results suggest that interoceptive attention might facilitate accuracy on discrimination tasks but that such attention was not particularly impacted by the training paradigm.

Only 1 unique effect of cognitive training was observed: participants in the cognitive training group demonstrated an increase in heart rate over time postpractice session but not for the prepractice session or pre- and postpractice in the MT group. This result may suggest that with an increased focus on negative symptoms during mood monitoring, participants in the cognitive training group may have experienced increased negative reactivity [89]. However, the cognitive training group did not demonstrate concurrent changes in mood or stress. Therefore, the results of this study may also suggest that as participants continued to play the cognitive training game, they may have become increasingly engaged with beating past performance and gaining a sense of achievement. It is not possible to conclude why postpractice heart rate was increasingly elevated for participants in the cognitive training group, but these results suggest that not all forms of physiological arousal are diagnostic of changes to mood or stress reactivity.

It is interesting that changes in heart rate were not observed for the MT group, especially as previous research has found decreases in heart rate following the completion of an 8-week mindfulness-based intervention [90]. However, this result highlights the fact that MT is not inherently *relaxing*. Instead, people may experience distress during MT as they initially approach difficult emotions, even if they experience less distress at the end of their practice [4], as observed in this study. Moreover, it has been shown that MT can concurrently decrease psychological distress and increase subjective energy levels [91]. Taken together, the results of this study suggest that changes in heart rate may not be required to reduce subjective stress levels.

### Limitations

Although this study provides evidence for the beneficial effects of MT using a smartphone app, there are several limitations that should be noted. First, studying app training inherently reduces the generalizability of findings to the richest segments of the global population. More specifically, our sample was limited to participants with Apple devices. Second, this study used a female-dominated sample, a factor that may also reduce generalizability. However, these limitations highlight the importance of replicating the present results across different operating devices and with a more diverse participant group. Third, although practice was monitored, participants were only reminded to practice if they missed 3 consecutive days. Therefore, participants did not necessarily practice with their assigned app (Wildflowers or 2048) every day, which might affect the extent of the significant findings observed. On the other hand, this limitation adds more ecological validity to this study as people in the real world would not be monitored closely to ensure they are practicing every day. Fourth, state mindfulness was not measured during daily training sessions, so it is hard to know if the benefits to mood and stress observed were a result of transiently increased state mindfulness or a result of another factor that was not considered in this study. However, a study design that promotes daily reflection on state mindfulness may have introduced further unintended training effects to the control group. Fifth, although the results strongly support benefits of MT on state measures of subjective well-being, the marginal pre- to postintervention results on the acceptance factor make it inappropriate to draw strong conclusions about the relative efficacy of MT relative to active control. These marginal results may be because of the power of this study or to the short intervention time of only 3 weeks. Although the *a priori* power analysis suggested adequate power, a post hoc simulation-based power analysis suggested that the study was underpowered for addressing these group by time interactions. Therefore, a future study with better power, and over a longer period, should attempt to replicate and extend our understanding of the relationship between the state and trait well-being factors. Sixth, it is possible that participants in the cognitive training group may have used their assigned app as a form of avoidance from daily stressors, which could have contributed to the increase in acceptance and awareness observed in this study. However, if participants were using the cognitive training app as a source of experiential avoidance, it would be expected that state stress ratings would have been reduced after a cognitive training session. Therefore, although it is not completely clear why changes in acceptance and awareness were observed in this group, it is more likely that these changes are related to increases in emotional self-awareness when recording mood and stress levels before each use of the app [79]. Finally, although the data were reduced with an exploratory factor analysis, a number of statistical models were still conducted to test each of the outcome variables. However, a binomial test was conducted, which indicated that the probability of finding the number of significant results observed in this study was low (*P*<.001; 95% CI 0.11-0.36).

### Future Directions

This study provides preliminary evidence on the benefits of using an MT smartphone app. These findings suggest that future work should continue to investigate the benefits of MT apps in clinical populations. In addition, future studies should investigate the longitudinal effects of using MT apps. Finally, the results of this study on improvements in attention regulation warrant studies exploring neural changes as a result of MT using a smartphone app. For example, Tang and colleagues observed that 2 weeks of brief mindfulness training altered the resting state functional connectivity of large-scale brain networks [92]. Therefore, it may be fruitful for future studies to explore both the self-reported, behavioral, and neural benefits of MT using a smartphone app.

### Conclusions

The results of this study suggest that MT with a smartphone app may provide immediate effects on mood and stress while also providing long-term benefits for attentional control. Although further investigation is warranted, there is evidence that with continued usage, MT via a smartphone app may provide long-term benefits in changing how one relates to his or her inner and outer experiences.

## Appendix

Multimedia Appendix 1 Screenshots of Wildflowers mindfulness training condition.

Multimedia Appendix 2 Screenshots of 2048 cognitive training control conditions.

Multimedia Appendix 3 Additional details and psychometric properties of the questionnaires.

Multimedia Appendix 4 Theoretical rationale for the newly developed respiration integration task.

Multimedia Appendix 5 Additional details for statistical analyses.

Multimedia Appendix 6 Scree plot of Horn’s Parallel Analysis of Principal Components used to determine the appropriate factor solution for the exploratory factor analysis.

Multimedia Appendix 7 Multilevel model figures for acceptance, awareness, openness, alerting, and orienting.

Multimedia Appendix 8 Multilevel model results for the individual questionnaire subscales.

Multimedia Appendix 9 CONSORT‐EHEALTH checklist (V 1.6.1).

## Abbreviations

AAQ-II: Acceptance and Action Questionnaire-II
ANT: Attention Network Test
BFI: Big Five Inventory
CRSD-ANT: Centre for Research on Safe Driving Attention Network Test
EFA: exploratory factor analysis
IBMT: integrated body-mind training
MAIA: Multidimensional Assessment of Interoceptive Awareness
MBCT: mindfulness-based cognitive therapy
MBSR: mindfulness-based stress reduction
MLQ: Meaning in Life Questionnaire
MT: mindfulness training
PHLMS: Philadelphia Mindfulness Scale
PSS: Perceived Stress Scale
PWBS: Psychological Well-Being Scale
REB: Research Ethics Board
RIT: Respiration Integration Task
SEI-R: Spiritual Experience Index-Revised

## References

[1] Brown KW, Ryan RM (2003). The benefits of being present: mindfulness and its role in psychological well-being. J Pers Soc Psychol.

[2] Kabat-Zinn J (1982). An outpatient program in behavioral medicine for chronic pain patients based on the practice of mindfulness meditation: theoretical considerations and preliminary results. Gen Hosp Psychiatry.

[3] Segal Z, Williams J, Teasdale J (2012). Mindfulness-based cognitive therapy for depression.

[4] Baer R (2003). Mindfulness training as a clinical intervention: a conceptual and empirical review. Clin Psychol Sci Pract.

[5] Kabat-Zinn J (2003). Mindfulness-based interventions in context: past, present, and future. Clin Psychol Sci Pract.

[6] Bishop S, Lau M, Shapiro S, Carlson L, Anderson N, Carmody J, Segal Z, Abbey S, Speca M, Velting D, Devins G (2004). Mindfulness: a proposed operational definition. Clin Psychol Sci Pract.

[7] Goyal M, Singh S, Sibinga EM, Gould NF, Rowland-Seymour A, Sharma R, Berger Z, Sleicher D, Maron DD, Shihab HM, Ranasinghe PD, Linn S, Saha S, Bass EB, Haythornthwaite JA (2014). Meditation programs for psychological stress and well-being: a systematic review and meta-analysis. JAMA Intern Med.

[8] Eberth J, Sedlmeier P (2012). The effects of mindfulness meditation: a meta-analysis. Mindfulness.

[9] Khoury B, Lecomte T, Fortin G, Masse M, Therien P, Bouchard V, Chapleau M, Paquin K, Hofmann SG (2013). Mindfulness-based therapy: a comprehensive meta-analysis. Clin Psychol Rev.

[10] Visted E, Vøllestad J, Nielsen MB, Nielsen GH (2014). The impact of group-based mindfulness training on self-reported mindfulness: a systematic review and meta-analysis. Mindfulness.

[11] Sedlmeier P, Eberth J, Schwarz M, Zimmermann D, Haarig F, Jaeger S, Kunze S (2012). The psychological effects of meditation: a meta-analysis. Psychol Bull.

[12] Creswell JD (2017). Mindfulness Interventions. Annu Rev Psychol.

[13] Keng S, Smoski MJ, Robins CJ (2011). Effects of mindfulness on psychological health: a review of empirical studies. Clin Psychol Rev.

[14] Bernstein A, Hadash Y, Lichtash Y, Tanay G, Shepherd K, Fresco DM (2015). Decentering and related constructs: a critical review and metacognitive processes model. Perspect Psychol Sci.

[15] Hayes SC, Wilson KG, Gifford EV, Follette VM, Strosahl K (1996). Experimental avoidance and behavioral disorders: a functional dimensional approach to diagnosis and treatment. J Consult Clin Psychol.

[16] Block-Lerner J, Wulfert E, Moses E (2009). ACT in context: an exploration of experiential acceptance. Cogn Behav Pract.

[17] Bond FW, Hayes SC, Baer RA, Carpenter KM, Guenole N, Orcutt HK, Waltz T, Zettle RD (2011). Preliminary psychometric properties of the Acceptance and Action Questionnaire-II: a revised measure of psychological inflexibility and experiential avoidance. Behav Ther.

[18] Kabat-Zinn J (1982). An outpatient program in behavioral medicine for chronic pain patients based on the practice of mindfulness meditation: theoretical considerations and preliminary results. Gen Hosp Psychiatry.

[19] Kabat-Zinn J (1990). Full Catastrophe Living: Using the Wisdom of Your Body and Mind to Face Stress, Pain and Illness.

[20] Fish J, Brimson J, Lynch S (2016). Mindfulness interventions delivered by technology without facilitator involvement: What research exists and what are the clinical outcomes?. Mindfulness (N Y).

[21] van Emmerik AA, Berings F, Lancee J (2018). Efficacy of a mindfulness-based mobile application: a randomized waiting-list controlled trial. Mindfulness (N Y).

[22] Manotas M, Segura C, Eraso M, Oggins J, McGovern K (2014). Association of brief mindfulness training with reductions in perceived stress and distress in Colombian health care professionals. IJSM.

[23] Zeidan F, Johnson SK, Diamond BJ, David Z, Goolkasian P (2010). Mindfulness meditation improves cognition: evidence of brief mental training. Conscious Cogn.

[24] Zeidan F, Johnson SK, Gordon NS, Goolkasian P (2010). Effects of brief and sham mindfulness meditation on mood and cardiovascular variables. J Altern Complement Med.

[25] Carmody J, Baer RA (2009). How long does a mindfulness-based stress reduction program need to be? A review of class contact hours and effect sizes for psychological distress. J Clin Psychol.

[26] Cavanagh K, Churchard A, O'Hanlon P, Mundy T, Votolato P, Jones F, Gu J, Strauss C (2018). A randomised controlled trial of a brief online mindfulness-based intervention in a non-clinical population: replication and extension. Mindfulness (N Y).

[27] Mikolasek M, Berg J, Witt CM, Barth J (2018). Effectiveness of mindfulness- and relaxation-based eHealth interventions for patients with medical conditions: a systematic review and synthesis. Int J Behav Med.

[28] Russell L, Ugalde A, Milne D, Austin D, Livingston P (2018). Digital characteristics and dissemination indicators to optimize delivery of internet-supported mindfulness-based interventions for people with a chronic condition: systematic review. JMIR Ment Health.

[29] Kvillemo P, Brandberg Y, Bränström R (2016). Feasibility and outcomes of an internet-based mindfulness training program: a pilot randomized controlled trial. JMIR Ment Health.

[30] Sliwinski J, Katsikitis M, Jones CM (2017). A review of interactive technologies as support tools for the cultivation of mindfulness. Mindfulness.

[31] Chittaro L, Vianello A (2016). Evaluation of a mobile mindfulness app distributed through on-line stores: a 4-week study. Int J Hum Comput Stud.

[32] Toivonen KI, Zernicke K, Carlson LE (2017). Web-based mindfulness interventions for people with physical health conditions: systematic review. J Med Internet Res.

[33] Spijkerman MP, Pots WT, Bohlmeijer ET (2016). Effectiveness of online mindfulness-based interventions in improving mental health: a review and meta-analysis of randomised controlled trials. Clin Psychol Rev.

[34] Nararro-Haro M, Hoffman H, Garcia-Palacios A, Sampaio M, Alhalabi W, Hall K, Linehan M (2016). The use of virtual reality to facilitate mindfulness skills training in dialectical behavioral therapy for borderline personality disorder: a case study. Front Psychol.

[35] Navarro-Haro MV, López-Del-Hoyo Y, Campos D, Linehan MM, Hoffman HG, García-Palacios A, Modrego-Alarcón M, Borao L, García-Campayo J (2017). Meditation experts try virtual reality mindfulness: a pilot study evaluation of the feasibility and acceptability of virtual reality to facilitate mindfulness practice in people attending a mindfulness conference. PLoS One.

[36] Flores A, Linehan M, Todd S, Hoffman H (2018). The use of virtual reality to facilitate mindfulness skills training in dialectical behavioral therapy for spinal cord injury: a case study. Front Psychol.

[37] Mani M, Kavanagh DJ, Hides L, Stoyanov SR (2015). Review and evaluation of mindfulness-based iPhone apps. JMIR Mhealth Uhealth.

[38] Plaza I, Demarzo MM, Herrera-Mercadal P, García-Campayo J (2013). Mindfulness-based mobile applications: literature review and analysis of current features. JMIR Mhealth Uhealth.

[39] Howells A, Ivtzan I, Eiroa-Orosa FJ (2014). Putting the ‘app’ in happiness: a randomised controlled trial of a smartphone-based mindfulness intervention to enhance wellbeing. J Happiness Stud.

[40] Economides M, Martman J, Bell MJ, Sanderson B (2018). Improvements in stress, affect, and irritability following brief use of a mindfulness-based smartphone app: a randomized controlled trial. Mindfulness (N Y).

[41] Bostock S, Crosswell AD, Prather AA, Steptoe A (2018). Mindfulness on-the-go: Effects of a mindfulness meditation app on work stress and well-being. J Occup Health Psychol.

[42] Fredrickson BL, Kahneman D (1993). Duration neglect in retrospective evaluations of affective episodes. J Pers Soc Psychol.

[43] Ryan RM, Deci EL (2001). On happiness and human potentials: a review of research on hedonic and eudaimonic well-being. Annu Rev Psychol.

[44] Bornemann B, Herbert BM, Mehling WE, Singer T (2014). Differential changes in self-reported aspects of interoceptive awareness through 3 months of contemplative training. Front Psychol.

[45] Fissler M, Winnebeck E, Schroeter T, Gummersbach M, Huntenburg JM, Gaertner M, Barnhofer T (2016). An investigation of the effects of brief mindfulness training on self-reported interoceptive awareness, the ability to decenter, and their role in the reduction of depressive symptoms. Mindfulness.

[46] Farb NA, Segal ZV, Anderson AK (2013). Mindfulness meditation training alters cortical representations of interoceptive attention. Soc Cogn Affect Neurosci.

[47] Fischer D, Messner M, Pollatos O (2017). Improvement of interoceptive processes after an 8-week body scan intervention. Front Hum Neurosci.

[48] Shearer A, Hunt M, Chowdhury M, Nicol L (2015). Effects of a brief mindfulness meditation intervention on student stress and heart rate variability internet. Int J Stress Manag.

[49] Tang Y, Ma Y, Wang J, Fan Y, Feng S, Lu Q, Yu Q, Sui D, Rothbart MK, Fan M, Posner MI (2007). Short-term meditation training improves attention and self-regulation. Proc Natl Acad Sci U S A.

[50] Cavanagh K, Strauss C, Cicconi F, Griffiths N, Wyper A, Jones F (2013). A randomised controlled trial of a brief online mindfulness-based intervention. Behav Res Ther.

[51] Chwyl B, Chung A, Amelard R, Deglint J, Clausi D, Wong A (2016). SAPPHIRE: Stochastically acquired photoplethysmogram for heart rate inference in realistic environments.

[52] Chwyl B, Chung A, Amelard R, Deglint J, Clausi D, Wong A (2016). Time-Frequency Domain Analysis via Pulselets for Non-contact Heart Rate Estimation from Remotely Acquired Photoplethysmograms.

[53] Chwyl B, Amelard R, Clausi D, Wong A (2016). A Bayesian multi-scale framework for photoplethysmogram imaging waveform processing. J Comput Vis Imaging Syst.

[54] Cohen S, Kamarck T, Mermelstein R (1983). A global measure of perceived stress. J Health Soc Behav.

[55] Mitchell AM, Crane PA, Kim Y (2008). Perceived stress in survivors of suicide: psychometric properties of the Perceived Stress Scale. Res Nurs Health.

[56] John O, Donahue E, Kentle R (1991). The Big Five Inventory--Versions 4a and 54.

[57] John OP, Srivastava S, Pervin LA, John OP (1999). The Big-Five trait taxonomy: History, measurement, theoretical perspectives. Handbook of personality: Theory and research (2nd ed.).

[58] Benet-Martínez V, John OP (1998). Los Cinco Grandes across cultures and ethnic groups: multitrait multimethod analyses of the Big Five in Spanish and English. J Pers Soc Psychol.

[59] Ryff CD, Keyes CL (1995). The structure of psychological well-being revisited. J Pers Soc Psychol.

[60] Ryff CD (1989). Happiness is everything, or is it? Explorations on the meaning of psychological well-being. J Pers Soc Psychol.

[61] Cardaciotto L, Herbert JD, Forman EM, Moitra E, Farrow V (2008). The assessment of present-moment awareness and acceptance: the Philadelphia Mindfulness Scale. Assessment.

[62] Park T, Reilly-Spong M, Gross CR (2013). Mindfulness: a systematic review of instruments to measure an emergent patient-reported outcome (PRO). Qual Life Res.

[63] Mehling WE, Price C, Daubenmier JJ, Acree M, Bartmess E, Stewart A (2012). The multidimensional assessment of interoceptive awareness (MAIA). PLoS One.

[64] Genia V (1991). The spiritual experience index: a measure of spiritual maturity. J Relig Health.

[65] Steger M, Frazier P, Oishi S, Kaler M (2006). The Meaning in Life Questionnaire: assessing the presence of and search for meaning in life. J Couns Psychol.

[66] Watson D, Clark LA, Tellegen A (1988). Development and validation of brief measures of positive and negative affect: the PANAS scales. J Pers Soc Psychol.

[67] Shacham S (1983). A shortened version of the profile of mood states. J Pers Assess.

[68] Västfjäll D, Friman M, Gärling T, Kleiner M (2002). The measurement of core affect: a Swedish self-report measure derived from the affect circumplex. Scand J Psychol.

[69] Weaver B, Bédard M, McAuliffe J (2013). Evaluation of a 10-minute version of the Attention Network Test. Clin Neuropsychol.

[70] Fan J, McCandliss BD, Sommer T, Raz A, Posner MI (2002). Testing the efficiency and independence of attentional networks. J Cogn Neurosci.

[71] R Core Team (2017). R Foundation for Statistical Computing.

[72] Cohen J (1988). Statistical power analysis for the behavioral sciences. 2nd ed.

[73] Dinno A (2012). https://cran.r-project.org/web/packages/paran/index.html.

[74] Revelle W (2016). http://cran.r-project.org/web/packages/psych/index.html.

[75] Pinheiro J, Bates D, DebRoy S, Sarkar D, R Core Team (2017). https://cran.r-project.org/web/packages/nlme/index.html.

[76] Lenth RV (2016). Least-Squares Means: the package. J Stat Soft.

[77] Lindsay EK, Young S, Smyth JM, Brown KW, Creswell JD (2018). Acceptance lowers stress reactivity: dismantling mindfulness training in a randomized controlled trial. Psychoneuroendocrinology.

[78] Shallcross AJ, Troy AS, Boland M, Mauss IB (2010). Let it be: Accepting negative emotional experiences predicts decreased negative affect and depressive symptoms. Behav Res Ther.

[79] Kauer S, Reid S, Crooke A, Khor A, Hearps S, Jorm A, Sanci L, Patton G (2012). Self-monitoring using mobile phones in the early stages of adolescent depression: randomized controlled trial. J Med Internet Res.

[80] van den Hurk PA, Wingens T, Giommi F, Barendregt HP, Speckens AE, van Schie HT (2011). On the relationship between the practice of mindfulness meditation and personality-an exploratory analysis of the mediating role of mindfulness skills. Mindfulness (N Y).

[81] Ainsworth B, Eddershaw R, Meron D, Baldwin DS, Garner M (2013). The effect of focused attention and open monitoring meditation on attention network function in healthy volunteers. Psychiatry Res.

[82] Moore A, Malinowski P (2009). Meditation, mindfulness and cognitive flexibility. Conscious Cogn.

[83] Hodgins HS, Adair KC (2010). Attentional processes and meditation. Conscious Cogn.

[84] Jha AP, Krompinger J, Baime MJ (2007). Mindfulness training modifies subsystems of attention. Cogn Affect Behav Neurosci.

[85] Wolkin JR (2015). Cultivating multiple aspects of attention through mindfulness meditation accounts for psychological well-being through decreased rumination. Psychol Res Behav Manag.

[86] Petersen SE, Posner MI (2012). The attention system of the human brain: 20 years after. Annu Rev Neurosci.

[87] Lyvers M, Makin C, Toms E, Thorberg FA, Samios C (2013). Trait mindfulness in relation to emotional self-regulation and executive function. Mindfulness.

[88] Teper R, Inzlicht M (2013). Meditation, mindfulness and executive control: the importance of emotional acceptance and brain-based performance monitoring. Soc Cogn Affect Neurosci.

[89] Dubad M, Winsper C, Meyer C, Livanou M, Marwaha S (2018). A systematic review of the psychometric properties, usability and clinical impacts of mobile mood-monitoring applications in young people. Psychol Med.

[90] Amutio A, Martínez-Taboada C, Hermosilla D, Delgado LC (2015). Enhancing relaxation states and positive emotions in physicians through a mindfulness training program: a one-year study. Psychol Health Med.

[91] Canby NK, Cameron IM, Calhoun AT, Buchanan GM (2014). A brief mindfulness intervention for healthy college students and its effects on psychological distress, self-control, meta-mood, and subjective vitality. Mindfulness.

[92] Tang Y, Tang Y, Tang R, Lewis-Peacock JA (2017). Brief mental training reorganizes large-scale brain networks. Front Syst Neurosci.

