# Supplementary figures and images for: Effects of a Mindfulness Meditation App on Subjective Well-Being: Active Randomized Controlled Trial and Experience Sampling Study
Source: JMIR Ment Health. 2019 Jan 8;6(1):e10844. doi: 10.2196/10844 (PMC6329416; doi:10.2196/10844)

Multimedia Appendix 1. Screenshots of Wildflowers mindfulness training condition.

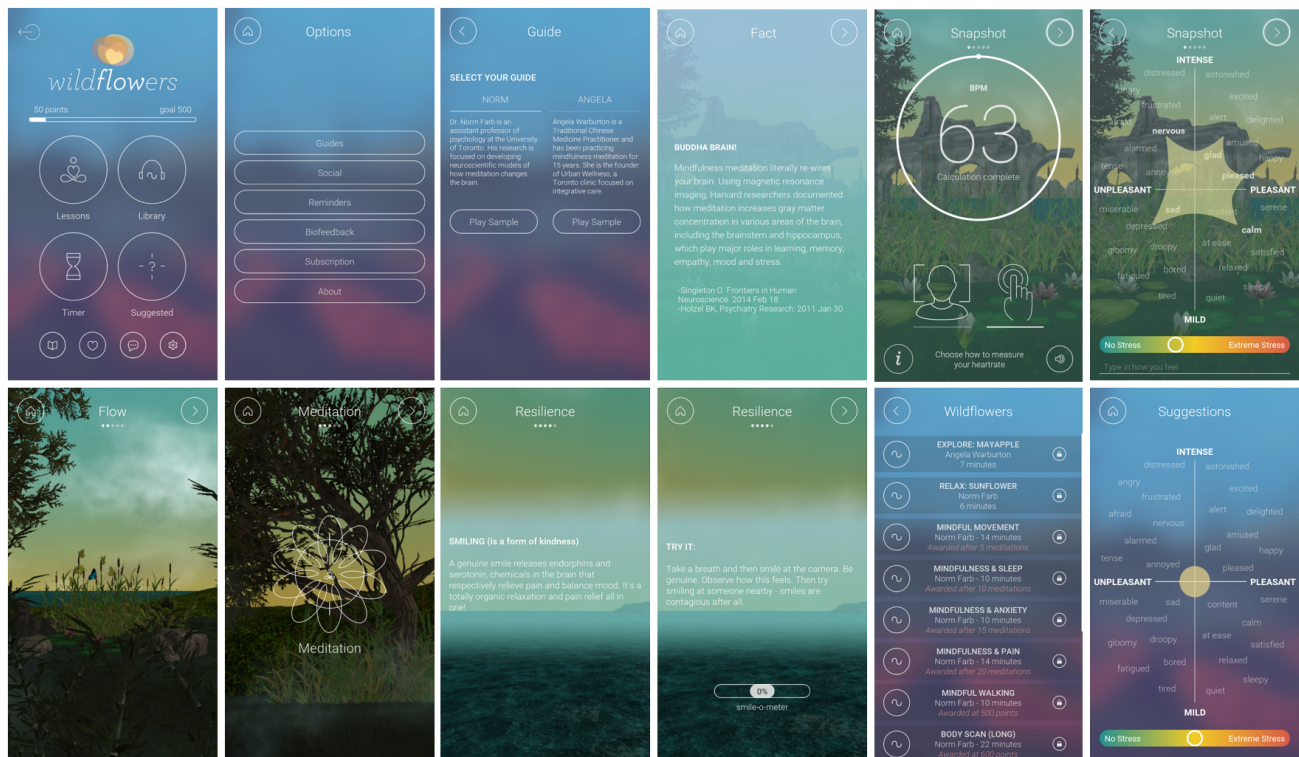

Supplement: Multimedia Appendix 1 [file mental_v6i1e10844_app1.pdf]

Multimedia Appendix 2. Screenshots of 2048 cognitive training control condition.

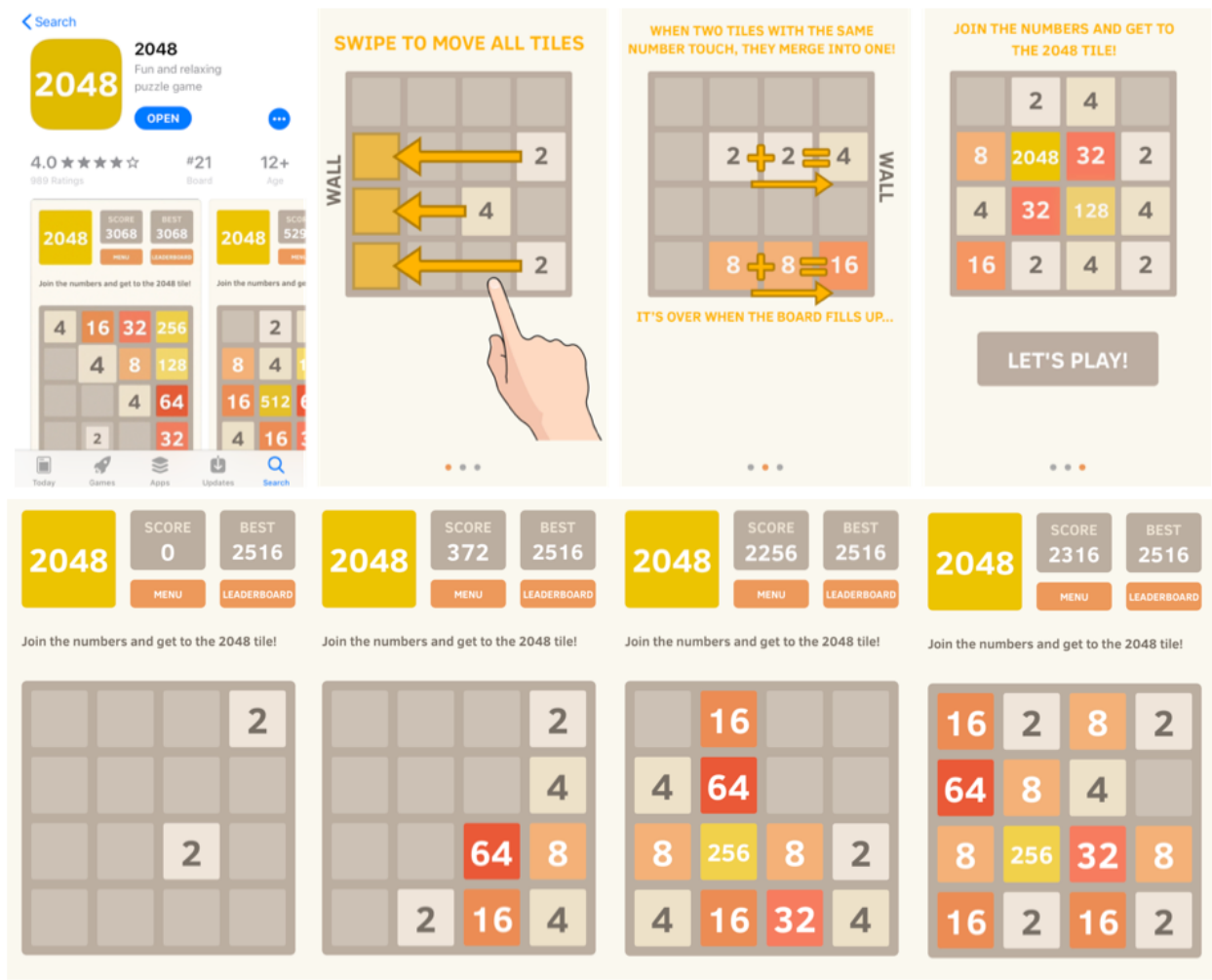

Supplement: Multimedia Appendix 2 [file mental_v6i1e10844_app2.pdf]
