# Supplementary material for: Effects of a Mindfulness Meditation App on Subjective Well-Being: Active Randomized Controlled Trial and Experience Sampling Study
Source: JMIR Ment Health. 2019 Jan 8;6(1):e10844. doi: 10.2196/10844 (PMC6329416; doi:10.2196/10844)
Supplement: Multimedia Appendix 3 [file mental_v6i1e10844_app3.pdf]

Multimedia Appendix 3. Additional details and psychometric properties of the questionnaires.

Perceived Stress Scale

The Perceived Stress Scale (PSS) [1] is a 10-item scale that measures the global perception of stress. However, because of a question that was inadvertently missing when the 10-item PSS questionnaire was loaded onto the survey platform, Qualtrics, participants from both groups did not see or respond to this missing question during data collection. Therefore, results from the short 4-item version of the PSS were alternatively used in subsequent analyses. Internal consistency has been reported as $\alpha = 0.82$ and convergent validity for the 4-item version has been reported as $r = 0.58$ to $r = 0.70$ [2]. Test retest evidence for the 4-item version is lacking; however, test retest reliability has been reported for the 10-item version as $r > 0.70$ [3].

Big Five Inventory

The Big Five Inventory (BFI) [4,5] is a 44-item scale that measures the 5 dimensions of personality. This scale contains five subscales that reflect the five dimensions of personality: extraversion, agreeableness, conscientiousness, neuroticism, and openness. Extraversion includes sociability, assertiveness, and positive emotionality. Agreeableness includes altruism, tender-mindedness, trust, and modesty. Conscientiousness includes thinking before acting, delaying gratification, following norms and rules, and organizational tendencies. Neuroticism includes anxiousness, nervousness, sadness, and tenseness. Lastly, openness contrasts openness to experience with close mindedness. Internal consistency has been reported as $\alpha = 0.75$ to $\alpha = 0.90$, test retest reliability has been reported as $r = 0.80$ to $r = 0.90$, and convergent validity has been reported as $r = 0.71$ to $r = 0.97$ [5,6].

Psychological Well-Being Scale

The Psychological Well-Being Scale (PWBS) [7] is an 84-item questionnaire that measures psychological well-being. This measure includes 6 subscales. The autonomy subscale contrasts self-determination and independence with concerns about the evaluation of others. The self-acceptance subscale contrasts a positive attitude towards the self, accepting both good and bad qualities, with being dissatisfied with the self and certain qualities. The positive relations with others subscale contrasts having warm, trusting, and satisfying relationships versus having few close and trusting relationships. The environmental mastery subscale contrasts having a sense of competence versus difficulties managing every day affairs. The purpose in life subscale contrasts a sense of direction with a sense of a lack of meaning in life. Finally, the personal growth subscale contrasts having a consistent feeling of continued development with a sense of personal stagnation. For the PWBS, internal consistency has been reported as $\alpha = 0.33$ to $\alpha = 0.56$ [7] and convergent validity has been reported as $r = 0.25$ to $r = 0.73$. During the development of the PWBS, which included 20-items per subscale, test-retest reliability was reported as $r = 0.81$ to $r = 0.85$ [8].

Acceptance and Action Questionnaire-II

The Acceptance and Action Questionnaire-II (AAQ-II) [9] is a 7-item scale that measures psychological inflexibility and experiential avoidance. The AAQ-II has demonstrated satisfactory evidence of internal consistency ($\alpha = 0.88$), test-retest reliability at three months ($r = 0.81$) and one year ($r = 0.79$), and convergent validity ($r = 0.25$ to $r = 0.97$) [9].

Philadelphia Mindfulness Scale

The Philadelphia Mindfulness Scale (PHLMS) [10] is a 20-item scale that measures 2 components of mindfulness. This scale consists of two subscales: the awareness subscale, which reflects a continuous monitoring of current internal and/or external experience, and the acceptance subscale, which reflects a non-judgemental attitude towards internal and/or external experiences. The PHLMS has demonstrated sufficient evidence of internal consistency ($\alpha = 0.81$ to $\alpha = 0.85$) and convergent validity ($r = 0.21$ to $r = 0.54$) [10]. However, test-retest reliability has not been reported [11].

Multidimensional Assessment of Interoceptive Awareness

The Multidimensional Assessment of Interoceptive Awareness (MAIA) [12] is a 32-item scale that measures the multidimensional construct of interoceptive body awareness. This scale is made up of 8 subscales: noticing, not distracting, not worrying, attention regulation, emotional awareness, self-regulation, body listening, and trusting. Noticing reflects the awareness of body sensations. Not-distracting reflects not avoiding sensations of pain or discomfort. Not-worrying reflects the tendency to not worry about psychological or physical discomfort. Attention regulation reflects the ability to sustain and control attention. Emotional awareness reflects awareness of the connection between physical sensations and psychological states. Self-regulation reflects the ability to regulate distress by attending to body sensations. Body listening reflects the ability to listen to the body. Lastly, trusting reflects the feeling that one's body is safe and able to be trusted. This scale has demonstrated sufficient evidence of convergent validity with other measures of body/mindful awareness, and internal consistency has been reported as $\alpha = 0.66$ to $\alpha = 0.82$ [12]. Evidence of test-retest reliability is lacking for the English version of the MAIA, however, has been reported for a German version of this test as $r = 0.66$ to $r = 0.79$ [13].

Spiritual Experience Index-Revised

The Spiritual Experience Index-Revised (SEI-R) [14] is a 23-item scale that measures a person's faith and spiritual journey. This scale consists of 2 subscales: the spiritual support subscale refers to the reliance on faith for support, and the spiritual openness subscale refers to being open to new spiritual experiences. The SEI-R has demonstrated sufficient evidence of convergent validity, and internal consistency has been reported as $\alpha = 0.79$ and $\alpha = 0.95$ [14]. However, evidence of test-retest reliability has not been reported.

Meaning in Life Questionnaire

The Meaning in Life Questionnaire (MLQ) [15] is a 10-item scale that measures 2 dimensions of the meaning in life and as such includes 2 subscales: presence of meaning, and search for meaning. Presence of meaning reflects the degree that a respondent feels that their life has meaning. Search for meaning reflects the degree that a respondent strives to find understanding in their life. Internal consistency has been reported as $\alpha = 0.82$ to $\alpha = 0.88$, test-retest reliability has been reported as $r = 0.70$ and $r = 0.73$, and convergent validity has been reported as $r = 0.28$ to $r = 0.74$ [15].

Mood Board Circumplex

The mood board is a visual representation of negative and positive emotions on a spectrum, ranging from intense emotions to mild emotions. This mood board provides a maximum of 32 emotions that a participant can select and yields 4 scores: degree of intense negative emotions,

degree of intense positive emotions, degree of mild negative emotions, and degree of mild positive emotions. This questionnaire is currently under validation; however, the words chosen for the mood board are commonly used in other measures of mood [16,17]. In addition, previous research has demonstrated the efficacy in taking these emotion-specific measures of mood and converting them to a visual analogue scale with 4 dimensions [18].

References

1. Cohen S, Kamarck T, Mermelstein R. A global measure of perceived stress. J Health Soc Behav 1983 Dec;24(4):385–96. PMID:6668417

2. Mitchell AM, Crane PA, Kim Y. Perceived stress in survivors of suicide: Psychometric properties of the Perceived Stress Scale. Res Nurs Health 2008 Dec;31(6):576–85. PMID:18449942

3. Lee E-H. Review of the psychometric evidence of the perceived stress scale. Asian Nurs Res 2012 Dec;6(4):121–127. PMID:25031113

4. John OP, Donahue EM, Kentle RL. The Big Five Inventory--Versions 4a and 54. Berkeley, CA: University of California, Berkeley, Institute of Personality and Social Research; 1991.

5. John OP, Srivastava S. The Big-Five trait taxonomy: History, measurement, and theoretical perspectives. In: Pervin LA, John OP, editors. Handb Personal Theory Res New York, NY: Guilford Press; 1999. 102–138.

6. Benet-Martínez V, John OP. Los Cinco Grandes across cultures and ethnic groups: multitrait multimethod analyses of the Big Five in Spanish and English. J Pers Soc Psychol 1998 Sep;75(3):729–50. PMID:9781409

7. Ryff CD, Keyes CL. The structure of psychological well-being revisited. J Pers Soc Psychol 1995 Oct;69(4):719–27. PMID:7473027

8. Ryff CD. Happiness is everything, or is it? Explorations on the meaning of psychological well-being. J Pers Soc Psychol 1989 Dec;57(6):1069–1081. [doi: 10.1037/0022-3514.57.6.1069]

9. Bond FW, Hayes SC, Baer RA, Carpenter KM, Guenole N, Orcutt HK, Waltz T, Zettle RD. Preliminary psychometric properties of the Acceptance and Action Questionnaire-II: a revised measure of psychological inflexibility and experiential avoidance. Behav Ther 2011 Dec;42(4):676–88. [doi: 10.1016/j.beth.2011.03.007]

10. Cardaciotto L, Herbert JD, Forman EM, Moitra E, Farrow V. The Assessment of Present-Moment Awareness and Acceptance: The Philadelphia Mindfulness Scale. Assessment 2008 Jun;15(2):204–23. PMID:18187399

11. Park T, Reilly-Spong M, Gross CR. Mindfulness: A systematic review of instruments to measure an emergent patient-reported outcome (PRO). Qual Life Res 2013 Dec;22(10):2639–59. PMID:23539467

12. Mehling WE, Price C, Daubenmier JJ, Acree M, Bartmess E, Stewart A. The multidimensional assessment of interoceptive awareness (MAIA). PLoS One 2012;7(11):e48230. PMID:23133619

13. Bornemann B, Herbert BM, Mehling WE, Singer T. Differential changes in self-reported aspects of interoceptive awareness through 3 months of contemplative training. Front Psychol 2014;5.

14. Genia V. The spiritual experience index: A measure of spiritual maturity. J Relig Health 1991 Dec;30(4):337–47. PMID:24272766

15. Steger MF, Frazier P, Oishi S, Kaler M. The Meaning in Life Questionnaire: Assessing the Presence of and Search for Meaning in Life. J Couns Psychol 2006;53(1):80–93.

16. Watson D, Clark LA, Tellegen A. Development and validation of brief measures of positive and negative affect: the PANAS scales. J Pers Soc Psychol 1988 Jun;54(6):1063–70. PMID:3397865

17. Shacham S. A shortened version of the Profile of Mood States. J Pers Assess 1983 Jun;47(3):305–6. PMID:6886962

18. Västfjäll D, Friman M, Gärling T, Kleiner M. The measurement of core affect: a Swedish self-report measure derived from the affect circumplex. Scand J Psychol 2002 Feb;43(1):19–31. PMID:11885757
