# Supplementary material for: Effects of a Mindfulness Meditation App on Subjective Well-Being: Active Randomized Controlled Trial and Experience Sampling Study
Source: JMIR Ment Health. 2019 Jan 8;6(1):e10844. doi: 10.2196/10844 (PMC6329416; doi:10.2196/10844)
Supplement: Multimedia Appendix 4 [file mental_v6i1e10844_app4.pdf]

Multimedia Appendix 4. Theoretical rationale for the newly developed respiration integration task.

The Respiration Integration Task (RIT)

The development of the RIT is motivated by our prior neuroimaging collaborations. In 2 studies, participants alternated the focus of attention between an external (visual) stimulus and internal (breath) stimulus while keeping their eyes open [1,2]. In the first of the studies (N=36), participants naïve to interoceptive training demonstrated the ability to recruit the posterior insula and surrounding somatosensory association cortex when attending to the breath relative to the visual stimulus [2]. The second study investigated the effects of interoceptive training through a mindfulness-based intervention [1]. Within the trained group (N=20), practice time reliably predicted the ability to recruit the posterior insula during internal (as compared to external) attention, $r(18)=0.61$, $P<.01$. Critically, the interoceptive region implicated in these studies, i.e., the long gyrus of the right posterior insula, is the same region associated with relapse prediction in our prior CIHR project. We therefore designed the RIT to contrast interoceptive attention to the breath against an external, vision-only baseline condition. To make task results interpretable outside of a neuroimaging context, we incorporated a behavioral response into the task to measure the effects of integrating breath sensation into judgments of stimulus duration.

The RIT Paradigm

The RIT employs a two-alternative forced choice paradigm, wherein participants watch a visually-presented circle pulse (expand and contract) twice. The first pulse serves as a reference stimulus, and participants are then asked to judge whether a second target pulse is faster or slower than the reference. The task involves 3 phases: 1) Visual Baseline: in each trial, participants' judgments are guided using vision alone; 2) Breath Entraining: participants' breathe along with the circle for 60 seconds to habituate to matching their respiration to the visual pulse cues; and 3) Breath Integration: participants breathe along with the pulses and then attempt change detection.

The aim of the task is to determine a person's Just Noticeable Difference threshold (JND) for detecting the respiratory change between the two pulses. In phases 1 & 3, a staircase method common to psychophysical investigations [3] is used to find each participant's JND threshold. The staircases follow a well-validated 1-Up/3-Down procedure [4]. In early trials, the differences between target and reference are large (~2 sec difference). Correct trials decrease the difference between target and reference, whereas incorrect trials increase the difference. A reversal – a change in direction from increasing difference to decreasing or vice versa - reflects a crossing of the participants' JND threshold. Every two reversals the step size decreases by half, narrowing around the specific boundary of the JND threshold. As recommended [4], 10 reversals are collected, with the last six reversals averaged to create JND scores. The goal of the task is to derive the specific ability to integrate interoceptive information, controlling for capacity using vision alone. Interoceptive function score is therefore calculated as:

RIT score=[JNDVisual Baseline]–[JNDBreath Integration]

Higher scores indicate adaptive integration of interoceptive sensation into the change detection task, analogous to the task demands of maintaining interoceptive awareness following dysphoric challenge.

RIT Validation

Extensive piloting with healthy undergraduates supports the RIT's potential.

Tolerability

In a first pilot of healthy undergraduate students (N=25), the task was universally well tolerated, and participants' ability to sync breathing with the circle pulses was excellent: objective measurement of respiratory phase was acquired using a respiration belt (Zephyr BioHarness 3) [5,6] revealing that the correlation between respiration and circle oscillations is nearly perfect ($r>0.95$).

Content Validity

A second study (N=100) supports the RIT's potential. A hierarchical regression approach assessed divergent (steps 1 & 2) and convergent (steps 3 & 4) validity:

- Step 1 controlled for demographic variables (age, gender) and respiration rate ($R2=0.046$, n.s.);
- Step 2 added performance on non-interoceptive psychophysics tasks including the Visual Baseline ($\Delta R2=0.068$, n.s.);
- Step 3 included the relationship with well-being (perceived stress [7], positive and negative affect [8], life satisfaction [9]) and found a significant association ($\Delta R2=.10$, $P<0.05$);
- Step 4 included self-reported body awareness (Private Body Consciousness [10], Multidimensional Assessment of Interoceptive Awareness- Noticing and Attention Regulation subscales [11], Philadelphia Mindfulness Inventory – Awareness subscale [12]) and found a marginally significant model improvement ($\Delta R2=0.08$, $P=0.07$).

In sum, the RIT shows validity as an indicator of well-being and interoceptive awareness, and is distinct from domain-general sensory acuity, affirming its suitability as an interoceptive marker.

References

1. Farb NA, Segal ZV, Anderson AK. Mindfulness meditation training alters cortical representations of interoceptive attention. Soc Cogn Affect Neurosci 2013;8(1):15–26. PMID:22689216

2. Farb NA, Segal ZV, Anderson AK. Attentional modulation of primary interoceptive and exteroceptive cortices. Cereb Cortex 2013 Jan;23(1):114–26. PMID: 22267308

3. Cornsweet TN. The Staircase-Method in Psychophysics. Am J Psychol 1962 Sep;75(3):485–491. [doi: 10.2307/1419876]

4. García-Pérez MA. Forced-choice staircases with fixed step sizes: asymptotic and small-sample properties. Vision Res 1998 Jun;38(12):1861–1881. PMID:9797963

5. Ainsworth B, Cahalin L, Buman M, Ross R. The Current State of Physical Activity Assessment Tools. Prog Cardiovasc Dis 2015;57(4):387–395. PMID:25446555

6. Johnstone JA, Ford PA, Hughes G, Watson T, Mitchell ACS, Garrett AT. Field Based Reliability and Validity of the Bioharness™ Multivariable Monitoring Device. J Sports Sci Med 2012 Dec;11(4):643–652. PMID:24150074

7. Cohen S, Kamarck T, Mermelstein R. A global measure of perceived stress. J Health Soc Behav 1983 Dec;24(4):385–96. PMID:6668417

8. Watson D, Clark LA, Tellegen A. Development and validation of brief measures of positive and negative affect: the PANAS scales. J Pers Soc Psychol 1988 Jun;54(6):1063–70. PMID:3397865

9. Diener E, Emmons RA, Larsen RJ, Griffin S. The Satisfaction With Life Scale. J Assess 1985 Feb;49(1):71–5. [doi: 10.1207/s15327752jpa4901_13]

10. Miller LC, Murphy R, Buss AH. Consciousness of body: Private and public. J Pers Soc Psychol 1981;41(2):397–406. [doi: 10.1037/0022-3514.41.2.397]

11. Mehling WE, Price C, Daubenmier JJ, Acree M, Bartmess E, Stewart A. The Multidimensional Assessment of Interoceptive Awareness (MAIA). PLOS ONE 2012 Nov;7(11):e48230. PMID:23133619

12. Cardaciotto L, Herbert JD, Forman EM, Moitra E, Farrow V. The Assessment of Present-Moment Awareness and Acceptance: The Philadelphia Mindfulness Scale. Assessment 2008 Jun 1;15(2):204–223. PMID:18187399
