# Supplementary material for: Effects of a Mindfulness Meditation App on Subjective Well-Being: Active Randomized Controlled Trial and Experience Sampling Study
Source: JMIR Ment Health. 2019 Jan 8;6(1):e10844. doi: 10.2196/10844 (PMC6329416; doi:10.2196/10844)
Supplement: Multimedia Appendix 5 [file mental_v6i1e10844_app5.pdf]

Multimedia Appendix 5. Additional details for statistical analyses.

Statistical Assumptions

Normality of residuals was examined through histograms of the residuals, which appeared to have relatively normal distributions. However, two possible outliers were identified, one in the orienting effect data and one in the circle task data. Therefore, influential cases were examined through calculating Cook's Distance [1] using the CookD function from the predict means package [2] in R. All of the Cook's Distances were less than 0.5, including the possible outliers identified in the orienting effect data and the circle task data, suggesting that there were no over influential cases in the data. Autocorrelation of residuals was calculated, and inspection of the autocorrelation plots revealed that the majority of the values in the data did not consistently depend on previous values, suggesting little autocorrelation. Lastly, homogeneity of variance was examined through inspection of residual versus fitted value scatterplots, which suggested no concerns with unequal variances.

Exploratory Factor Analysis: Factorability of Questionnaire Data

A correlation matrix (Table 1) revealed that each of the subscales, except for MLQ search for meaning, MAIA not worrying, and Negative Low subscales, correlated with at least one other subscale at 0.3 or greater. The Kaiser-Meyer-Olkin [3] test of sampling adequacy was 0.85, above the recommended value of .6, and Bartlett's test of sphericity [4] was significant ($\chi2$ (465)=1626.35, $p<0.001$. These results suggest the suitability of conducting an exploratory factor analysis that includes all of the subscales [5].

Table 1. Correlations between the self-report questionnaire subscales

	A	B	C	D	E	F	G	H	I	J	K	L	M	N	O	P	Q	R	S	T	U	V	W	X	Y	Z	AA	BB	CC	DD	EE
A. PSS Short	1	-0.22	-0.23	-0.32	0.53	-0.1	-0.36	-0.68	-0.24	-0.21	-0.45	-0.6	-0.52	-0.13	-0.51	-0.07	-0.29	-0.21	-0.24	-0.06	-0.22	-0.16	-0.36	0.12	-0.27	-0.39	0.18	0.29	0.38	-0.35	-0.24
B. BFI Extraversion		1	0.15	0.27	-0.32	0.28	0.35	0.31	0.38	0.37	0.46	0.44	0.31	0.17	0.21	0.16	0.15	0.24	0.19	0.29	0.21	0.15	0.35	0.16	0.19	0.3	0.11	-0.22	-0.23	0.17	0.04
C. BFI Agreeableness			1	0.19	-0.37	0.21	0.21	0.4	0.24	0.39	0.29	0.36	0.34	0.25	0.02	0.28	0.02	0.13	0.26	0.25	0.33	0.22	0.35	0.23	0.2	0.36	0.07	-0.16	-0.13	0.17	0.09
D. BFI Conscientiousness				1	-0.19	0.31	0.4	0.61	0.27	0.28	0.56	0.42	0.3	0.2	0.17	0.16	0.23	0.21	0.24	0.13	0.12	0.12	0.31	0.14	0.19	0.35	-0.14	0	-0.08	0.27	0.14
E. BFI Neuroticism					1	-0.13	-0.44	-0.49	-0.19	-0.29	-0.34	-0.51	-0.55	-0.03	-0.48	-0.05	-0.15	-0.37	-0.22	0.05	-0.19	-0.12	-0.37	0	-0.14	-0.25	0.28	0.33	0.33	-0.18	-0.17
F. BFI Openness						1	0.34	0.22	0.42	0.12	0.21	0.08	-0.03	0.41	-0.1	0.28	-0.1	0.17	0.36	0.4	0.36	0.24	0.35	0.06	0.32	0.14	0.16	0.12	0.05	0.32	0.26
G. PWBS Autonomy							1	0.49	0.41	0.28	0.5	0.55	0.48	0.13	0.27	0.17	0.16	0.43	0.38	0.17	0.26	0.19	0.46	-0.02	0.25	0.3	-0.22	-0.02	-0.1	0.28	0.28
H. PWBS Environmental Mastery								1	0.43	0.45	0.69	0.74	0.62	0.22	0.41	0.13	0.25	0.32	0.34	0.16	0.27	0.21	0.51	0.04	0.37	0.49	-0.17	-0.21	-0.35	0.3	0.19
I. PWBS Personal Growth									1	0.39	0.58	0.42	0.27	0.35	0.02	0.24	-0.06	0.16	0.23	0.33	0.3	0.17	0.38	0.04	0.43	0.18	0.19	0.09	0	0.36	0.24
J. PWBS Positive Relations										1	0.57	0.51	0.48	0.23	0.24	0.2	0.14	0.15	0.28	0.23	0.23	0.16	0.47	0.24	0.26	0.4	0.01	-0.12	-0.23	0.05	0.04
K. PWBS Purpose in Life											1	0.69	0.57	0.2	0.28	0.19	0.22	0.17	0.24	0.28	0.26	0.18	0.48	0.24	0.28	0.57	-0.08	-0.15	-0.28	0.25	0.1
L. PWBS Self Acceptance												1	0.76	0.14	0.51	0.1	0.29	0.28	0.24	0.12	0.23	0.11	0.49	0.12	0.36	0.54	-0.18	-0.22	-0.37	0.31	0.21
M. AAQ Total													1	0.02	0.62	0.03	0.37	0.31	0.25	-0.02	0.18	0.09	0.48	0.06	0.26	0.48	-0.28	-0.3	-0.35	0.15	0.12
N. PHLMS Awareness														1	-0.12	0.66	-0.05	0.01	0.54	0.62	0.46	0.4	0.35	0.04	0.2	0.11	0.19	0.17	0	0.27	0.24
O. PHLMS Acceptance															1	-0.12	0.49	0.28	0.09	-0.15	-0.05	-0.01	0.21	-0.18	0.2	0.27	-0.36	-0.2	-0.33	0.03	0.02
P. MAIA Noticing																1	-0.03	-0.07	0.6	0.68	0.54	0.48	0.45	0.03	0.17	0.05	0.22	0	-0.06	0.12	0.08
Q. MAIA Not Distracting																	1	0.06	0.07	0.01	-0.1	0.03	0.13	-0.03	0.08	0.35	-0.17	-0.2	-0.23	-0.08	-0.16
R. MAIA Not Worrying																		1	0.27	-0.05	0.1	0	0.11	-0.22	0.09	0.09	-0.13	-0.07	-0.08	0.17	0.16
S. MAIA Attention Regulation																			1	0.62	0.63	0.58	0.53	0.05	0.17	0.18	0.09	-0.09	-0.14	0.26	0.26
T. MAIA Emotional Awareness																				1	0.61	0.55	0.49	0.11	0.28	0.18	0.31	0.03	-0.02	0.2	0.15
U. MAIA Self-Regulation																					1	0.56	0.51	0.14	0.22	0.2	0.11	-0.09	-0.07	0.3	0.31
V. MAIA Body Listening																						1	0.47	0.15	0.17	0.17	0.26	-0.1	-0.03	0.18	0.26
W. MAIA Trusting																							1	0.24	0.4	0.48	0.06	-0.12	-0.2	0.27	0.22
X. SEI Support																								1	-0.01	0.41	0.03	-0.01	-0.05	0.07	0.02
Y. SEI Openness																									1	0.16	0.18	0.04	0.04	0.31	0.29
Z. MLQ Presence of Meaning																										1	-0.18	-0.22	-0.31	0.1	0.01
AA. MLQ Search for Meaning																											1	0.06	0.17	0.04	-0.01
BB. Intense Negative																												1	0.59	0.24	0.18
CC. Mild Negative																													1	0.08	0.19
DD. Intense Positive																														1	0.57
EE. Mild Positive																															1

References

1. Cook RD. Detection of Influential Observation in Linear Regression. Technometrics 1977;19(1):15–18. [doi: 10.2307/1268249]

2. Luo D, Ganesh S, Koolaard J. predictmeans: Calculate Predicted Means for Linear Models. R package version 0.99. 2014. Available from: https://CRAN.R-project.org/package=predictmeans

3. Kaiser HF, Rice J. Little Jiffy, Mark Iv. Educ Psychol Meas 1974 Apr;34(1):111–117. [doi: 10.1177/001316447403400115]

4. Snedecor GW, Cochran WG. Statistical Methods. 8th ed. Iowa State University Press; 1989.

5. Tabachnick BG, Fidell LS. Using Multivariate Statistics 5th ed. Needham Heights, MA, USA: Allyn & Bacon, Inc.; 2006. ISBN:978-0-205-45938-4
