# Supplementary material for: Effects of a Mindfulness Meditation App on Subjective Well-Being: Active Randomized Controlled Trial and Experience Sampling Study
Source: JMIR Ment Health. 2019 Jan 8;6(1):e10844. doi: 10.2196/10844 (PMC6329416; doi:10.2196/10844)

Multimedia Appendix 6. Scree plot of Horn's Parallel Analysis of Principal Components used to determine the appropriate factor solution for the exploratory factor analysis.

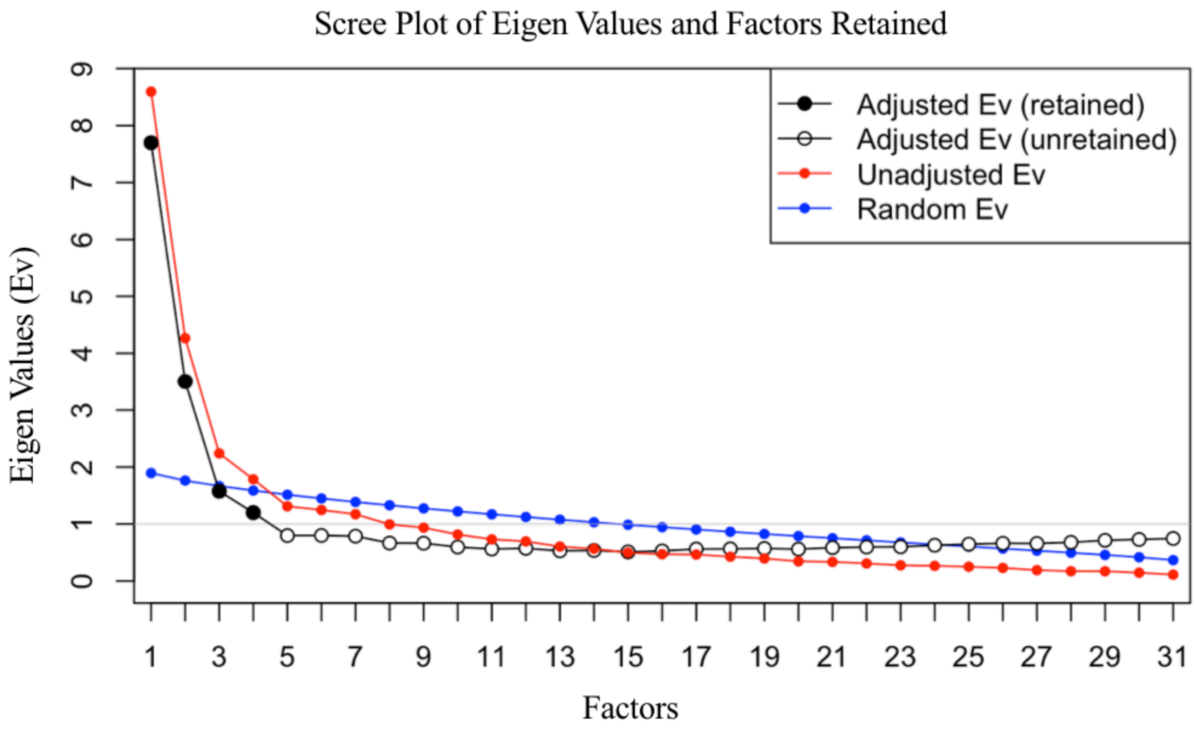

Supplement: Multimedia Appendix 6 [file mental_v6i1e10844_app6.pdf]
