# Supplementary material for: Effects of a Mindfulness Meditation App on Subjective Well-Being: Active Randomized Controlled Trial and Experience Sampling Study
Source: JMIR Ment Health. 2019 Jan 8;6(1):e10844. doi: 10.2196/10844 (PMC6329416; doi:10.2196/10844)

Multimedia Appendix 7. Multilevel model figures for acceptance, awareness, openness, alerting, and orienting.

Figure 1. Changes in acceptance before and after MT and cognitive training. MT: mindfulness training.

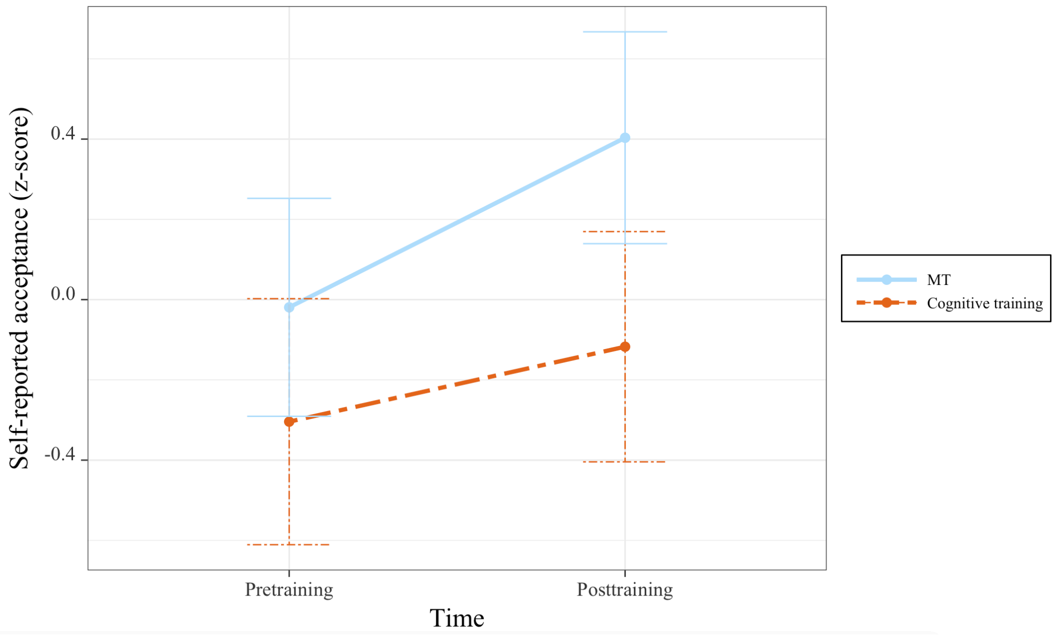

Figure 2. Changes in awareness before and after MT and cognitive training. MT: mindfulness training.

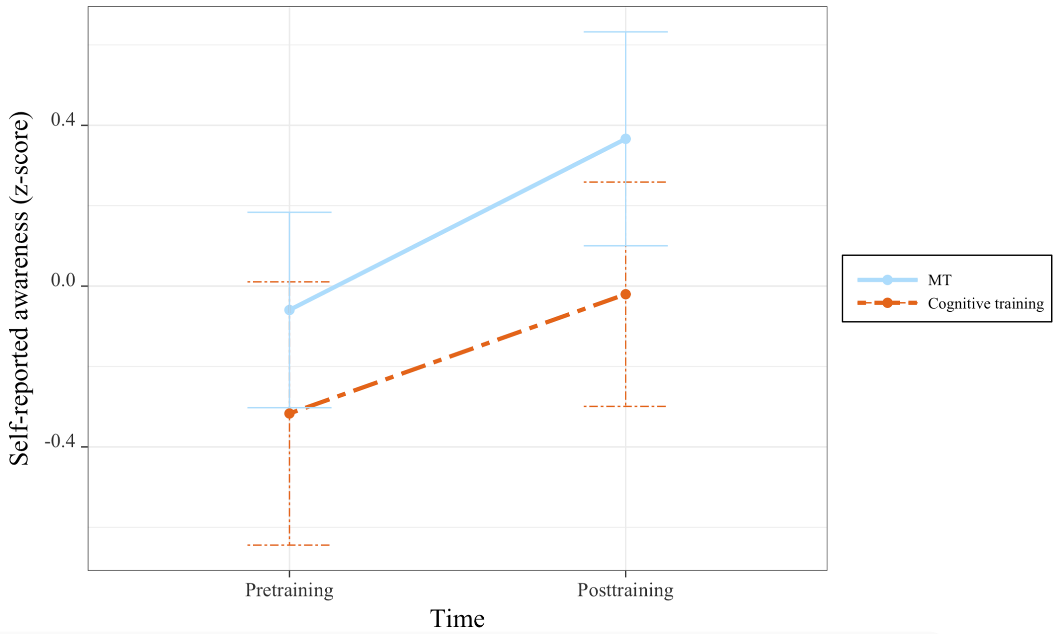

Figure 3. Changes in openness before and after MT and cognitive training. MT: mindfulness training.

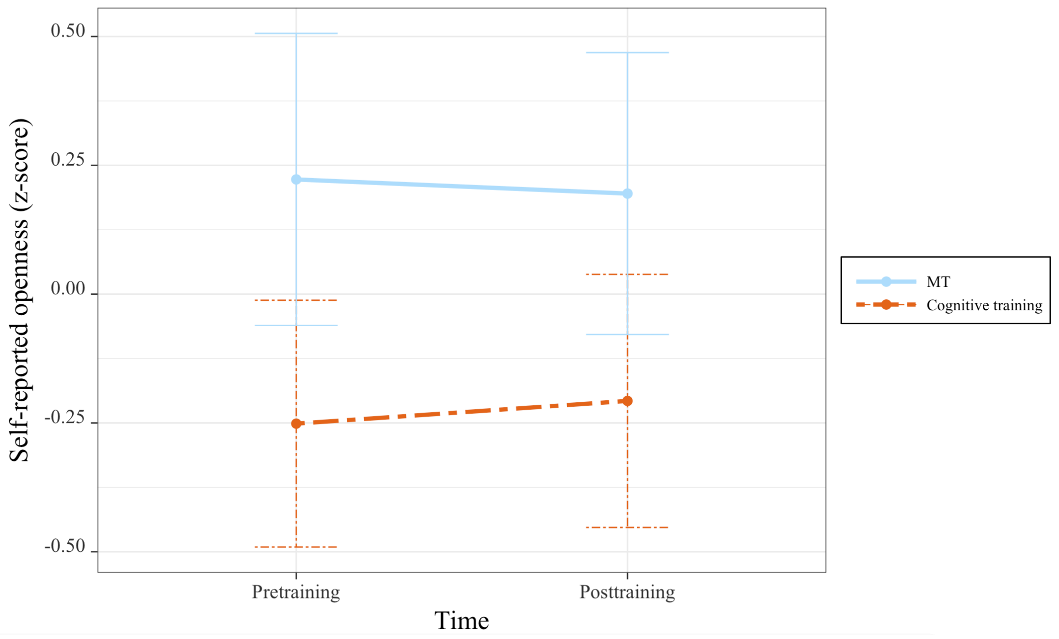

Figure 4. Changes in alerting effect before and after MT and cognitive training. MT: mindfulness training.

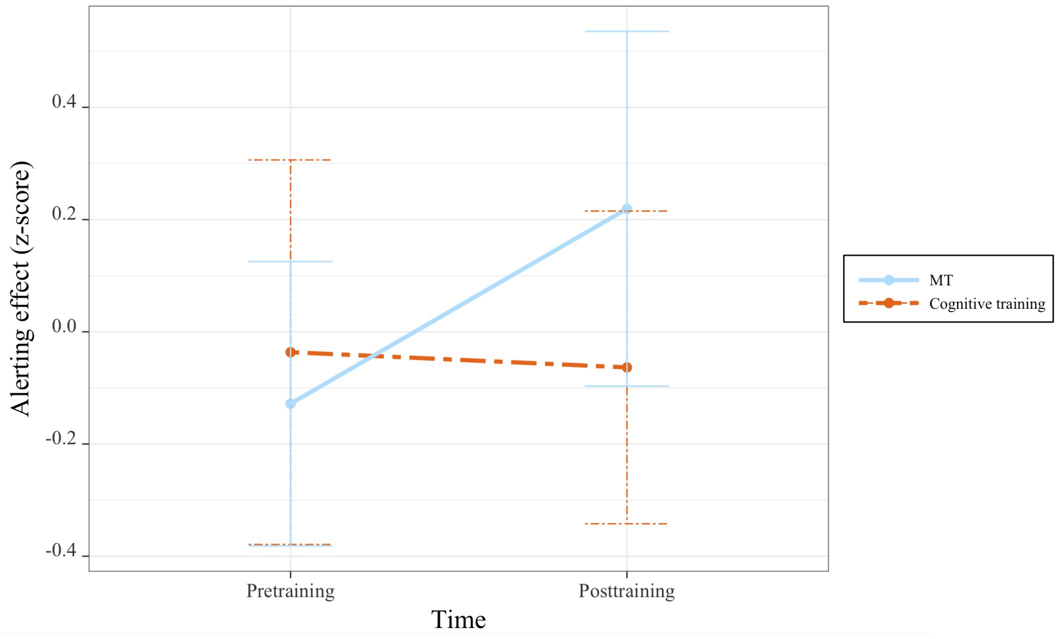

Figure 5. Changes in orienting effect before and after MT and cognitive training. MT: mindfulness training.

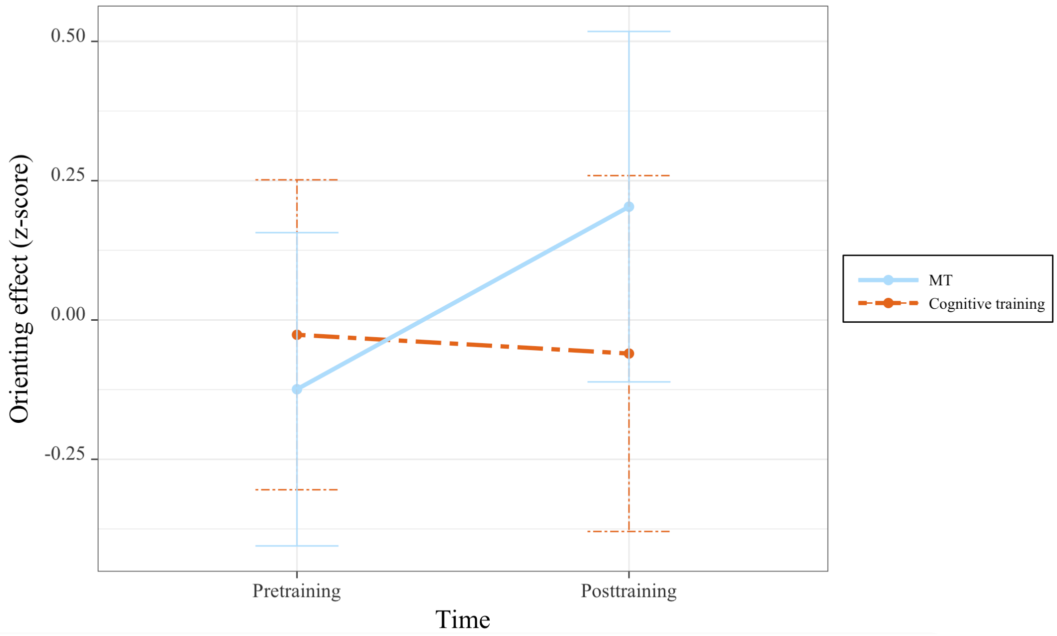

Supplement: Multimedia Appendix 7 [file mental_v6i1e10844_app7.pdf]
