# Supplementary material for: Effects of a Mindfulness Meditation App on Subjective Well-Being: Active Randomized Controlled Trial and Experience Sampling Study
Source: JMIR Ment Health. 2019 Jan 8;6(1):e10844. doi: 10.2196/10844 (PMC6329416; doi:10.2196/10844)
Supplement: Multimedia Appendix 8 [file mental_v6i1e10844_app8.pdf]

Multimedia Appendix 8. Multilevel model results for the individual questionnaire subscales.

Table 1. Multilevel models of trait measures of well-being.

Scale/subscale	Mindfulness training		Cognitive training		Group by time *P* value
	Mean pre score	Mean post score	Mean pre score	Mean post score	
PSS[a]-short version	6.51	5.44	8.20	7.02	.87
BFI[b]/Extraversion	24.96	25.42	23.40	24.61	.31
BFI/Agreeableness	34.11	34.91	34.85	34.76	.21
BFI/Conscientiousness	32.04	32.91	28.78	28.61	.11
BFI/Neuroticism	24.96	24.16	26.37	25.73	.82
BFI/Openness	35.56	36.04	34.73	34.78	.60
PWBS[c]/Autonomy	55.87	59.07	53.95	55.15	.20
PWBS/Environmental Mastery	55.20	58.89	48.63	52.27	.97
PWBS/Personal Growth	68.51	69.58	67.66	67.51	.47
PWBS/Positive Relations with Others	58.60	60.47	58.58	59.85	.69
PWBS/Purpose in Life	59.40	63.04	55.39	56.93	.28
PWBS/Self-Acceptance	56.47	60.27	53.66	54.17	**.09**[j]
AAQ-II[d]	25.24	27.82	25.85	25.02	**.03**[i]
PHLMS[e]/Awareness Subscale	32.98	33.36	31.07	31.56	.92
PHLMS/Acceptance Subscale	26.58	29.04	26.41	27.12	.16
MAIA[f]/Noticing	3.79	3.86	3.43	3.51	.97
MAIA/Not Distracting	2.71	2.90	2.74	2.76	.54
MAIA/Not Worrying	3.15	3.11	2.77	2.96	.30
MAIA/Attention Regulation	3.04	3.72	2.89	3.22	.13
MAIA/Emotional Awareness	3.85	4.14	3.44	3.91	.46
MAIA/Self-Regulation	2.95	3.95	2.99	3.42	**.04**[i]
MAIA/Body Listening	2.34	3.27	2.24	2.50	**.04**[i]
MAIA/Trusting	3.90	4.53	3.50	3.89	.36
SEI-R[g]/Support	45.04	47.87	47.95	49.66	.62
SEI-R/Openness	41.24	41.80	40.71	42.15	.42
MLQ[h]/Presence of Meaning	21.64	23.98	21.17	22.22	.24
MLQ/Search for Meaning	24.71	24.91	25.29	26.15	.66
Mood Board/Intense Negative Emotions	2.60	1.33	2.34	2.12	**.02**[i]

Mood Board/Mild Negative Emotions	2.38	1.62	2.78	2.02	>.99
Mood Board/Intense Positive Emotions	2.82	2.69	1.44	1.59	.50
Mood Board/Mild Positive Emotions	2.93	3.49	2.10	2.41	.65

[a]PSS: Perceived Stress Scale.

[b]BFI: Big Five Inventory.

[c]PWBS: Psychological Well-Being Scale.

[d]AAQ-II: Acceptance and Action Questionnaire.

[e]PHLMS: Philadelphia Mindfulness Scale.

[f]MAIA: Multidimensional Assessment of Interoceptive Awareness.

[g]SEI-R: Spiritual Experience Index.

[h]MLQ: Meaning in Life Questionnaire.

[i]Represents significant findings

[j]Represents marginal findings
